# Glycan strand cleavage by a lytic transglycosylase, MltD contributes to the expansion of peptidoglycan in *Escherichia coli*

**DOI:** 10.1371/journal.pgen.1011161

**Published:** 2024-02-29

**Authors:** Moneca Kaul, Suraj Kumar Meher, Krishna Chaitanya Nallamotu, Manjula Reddy

**Affiliations:** 1 CSIR-Centre for Cellular and Molecular Biology, Hyderabad, India; 2 Academy of Scientific and Innovative Research (AcSIR), Ghaziabad, India; Michigan State University, UNITED STATES

## Abstract

Peptidoglycan (PG) is a protective sac-like exoskeleton present in most bacterial cell walls. It is a large, covalently crosslinked mesh-like polymer made up of many glycan strands cross-bridged to each other by short peptide chains. Because PG forms a continuous mesh around the bacterial cytoplasmic membrane, opening the mesh is critical to generate space for the incorporation of new material during its expansion. In *Escherichia coli*, the ‘space-making activity’ is known to be achieved by cleavage of crosslinks between the glycan strands by a set of redundant PG endopeptidases whose absence leads to rapid lysis and cell death. Here, we demonstrate a hitherto unknown role of glycan strand cleavage in cell wall expansion in *E*. *coli*. We find that overexpression of a membrane-bound lytic transglycosylase, MltD that cuts the glycan polymers of the PG sacculus rescues the cell lysis caused by the absence of essential crosslink-specific endopeptidases, MepS, MepM and MepH. We find that cellular MltD levels are stringently controlled by two independent regulatory pathways; at the step of post-translational stability by a periplasmic adaptor-protease complex, NlpI-Prc, and post-transcriptionally by RpoS, a stationary-phase specific sigma factor. Further detailed genetic and biochemical analysis implicated a role for MltD in cleaving the nascent uncrosslinked glycan strands generated during the expansion of PG. Overall, our results show that the combined activity of PG endopeptidases and lytic transglycosylases is necessary for successful expansion of the cell wall during growth of a bacterium.

## Author summary

Most bacteria are protected by a cell wall made up of peptidoglycan (PG), a mesh-like large polymer. PG consists of several linear glycan strands interlinked through short peptide chains to form a continuous meshwork around the bacterial cytoplasmic membrane. Because PG tightly encases the cytoplasmic membrane, the growth of a bacterial cell is coupled to the expansion of PG requiring the activity of hydrolytic enzymes that cleave PG crosslinks to make space for incorporation of new PG material. ln *E*. *coli*, a set of redundant crosslink-specific endopeptidases are known to be crucial for expansion of PG. In this study, we show that cleavage of the glycan polymers by MltD compensates for the absence of crosslink cleavage and contributes to the expansion of PG. Overall, our work shows a previously unknown role of glycan hydrolases in cell wall expansion identifying these as potential targets for development of cell wall-specific antimicrobial agents.

## Introduction

Most bacteria are surrounded by a protective exoskeleton, peptidoglycan (PG or murein) that protects cells from lysis by internal osmotic pressure and harsh environmental conditions. In Gram-negative bacteria, PG is located in the periplasmic space in between the inner membrane (IM) that encloses the cytoplasm and a surface-exposed outer membrane (OM). PG is a covalently closed, mesh-like macromolecule that closely encases the IM providing shape to the bacterial cells. Structurally, it is made up of several linear glycan polymers containing repeating disaccharide units of N-acetylglucosamine (GlcNAc) and N-acetylmuramic acid (MurNAc) residues covalently bonded by β-1,4 glycosidic linkage. Each MurNAc residue of the glycans is attached to a short peptide chain consisting of, L-alanine^1^-D-glutamate^2^-meso-diaminopimelic acid (mDAP)^3^-D-alanine (D-ala)^4^-D-ala^5^. Approximately 40% of the peptide chains in the PG are crosslinked to each other; of these, approximately 90–93% form between the D-ala^4^ of one peptide chain and the mDAP^3^ of the adjacent chain (D-ala-mDAP or 4,3 crosslinks) by the catalytic activity of either class A (PBP1A, PBP1B) or class B (PBP2, PBP3) PG synthases whereas a small fraction (7–10%) form between two mDAP residues (mDAP^3^-mDAP^3^ or 3,3 crosslinks) by the activity of L,D-transpeptidases, LdtD and LdtE [1–4].

Because the PG forms a continuous mesh-like network around the IM, the growth and enlargement of a bacterial cell is tightly coupled to the expansion of PG sacculus [5]. In several Gram-negative bacteria, cleavage of crosslinks by PG endopeptidases has been shown to be essential for their growth and viability suggesting that opening the mesh by crosslink hydrolysis is fundamental to generate space for the incorporation of incoming glycan strands during PG expansion [6–8]. *E*. *coli* encodes several 4,3 crosslink specific PG hydrolases (termed D,D-endopeptidases) which include, MepA (*mepA*), MepS (*mepS*), MepM (*mepM*), MepH (*mepH*), Pbp4 (*dacB*), Pbp7 (*pbpG*) and AmpH (*ampH*) [6,9,10]. Among these, absence of MepS, -M and -H leads to rapid lysis and cell death demonstrating their essentiality at normal physiological growth conditions [6,11].

Although, crosslink hydrolysis is crucial for the expansion of PG, it needs to be controlled stringently as unfettered cleavage might lead to lethal degradation and rupture of the PG sacculus [12]. We previously showed that MepS, a key elongation-specific endopeptidase, is controlled at the step of post-translational stability by a periplasmic proteolytic system comprising an OM lipoprotein, NlpI and a soluble C-terminal specific protease, Prc [12]. Here, NlpI serves as an adaptor protein to bind both MepS and Prc to bring them into proximity to facilitate degradation of MepS by Prc [12,13]. The NlpI-Prc system is also conserved in *Pseudomonas aeruginosa* in which the homologs of MepS and MepM are cleaved by the protease CtpA through a lipoprotein adaptor LbcA [14–16].

In addition to endopeptidases, *E*. *coli* encodes several classes of PG hydrolases including carboxypeptidases, lytic transglycosylases and amidases which function in PG maturation, remodelling, turnover and daughter cell separation [9,10,17]. The lytic transglycosylases or LTGs catalyse the nonhydrolytic cleavage of β,1–4 glycosidic linkages between MurNAc and GlcNAc residues of PG with concomitant formation of a cyclic1,6- anhydro ring at the MurNAc residue [17–19]. Due to this distinct catalytic activity of LTGs, the termini of most glycan strands in *E*. *coli* contain 1,6- anhydro MurNAc residues. LTGs are believed to play a predominant role in PG recycling as the 1,6 anhydro muropeptides generated by their activity are transported through AmpG, an IM-permease to be further utilised for the synthesis of PG precursors in the cytosol [20].

Based on the substrate specificity, LTGs are either endolytic (cleaving within the glycan strand) or exolytic (cleaving from the ends of a glycan chain) [17,18]. *E*. *coli* encodes eight LTGs, of which MltA, -B, -C, -D, -E, -F are OM-associated; Slt is soluble periplasmic; and MltG is IM-anchored [17–19, 21]. In addition, a division-associated glycosyl hydrolase, DigH which specifically targets denuded glycan strands at the septum is known [22]. It is not yet clear why *E*. *coli* has such a large repertoire of glycan hydrolases; however, evidence suggests that they may possess distinct substrate-specificity to function in discrete PG pathways [21–23]. Interestingly, recent studies done in *Vibrio cholerae* propose an essential role for LTGs in cleaving the uncrosslinked glycan strands generated during PG synthesis to prevent their toxic crowding in the periplasm [24,25].

In this study, we show that glycan strand cleavage by an LTG, MltD [26] contributes to the process of PG expansion in *E*. *coli*. During an attempt to understand the mechanism of PG enlargement, we observed that overexpression of MltD, an OM lipoprotein compensates the absence of crosslink cleaving endopeptidases, MepS, MepM and MepH. Interestingly, cellular MltD abundance is maintained by two regulatory systems. It is controlled at the step of post-translational stability by NlpI-Prc mediated proteolysis and in addition, the MltD abundance in the stationary-phase cells is governed by the stationary-phase specific sigma factor, RpoS. Biochemical analysis shows MltD is an LTG that prefers uncrosslinked glycan strands. Notably, our results suggest that MltD activity contributes to optimal PG expansion by mitigating the deleterious defects arising due to PG synthesis which are exacerbated during endopeptidase insufficiency. To summarize, our study shows that MltD, a stringently controlled LTG has a role in PG enlargement during envelope biogenesis in *E*. *coli*.

## Results

### Absence of NlpI-Prc proteolytic machinery compensates the endopeptidase insufficiency in E. coli

**Fig 1:**
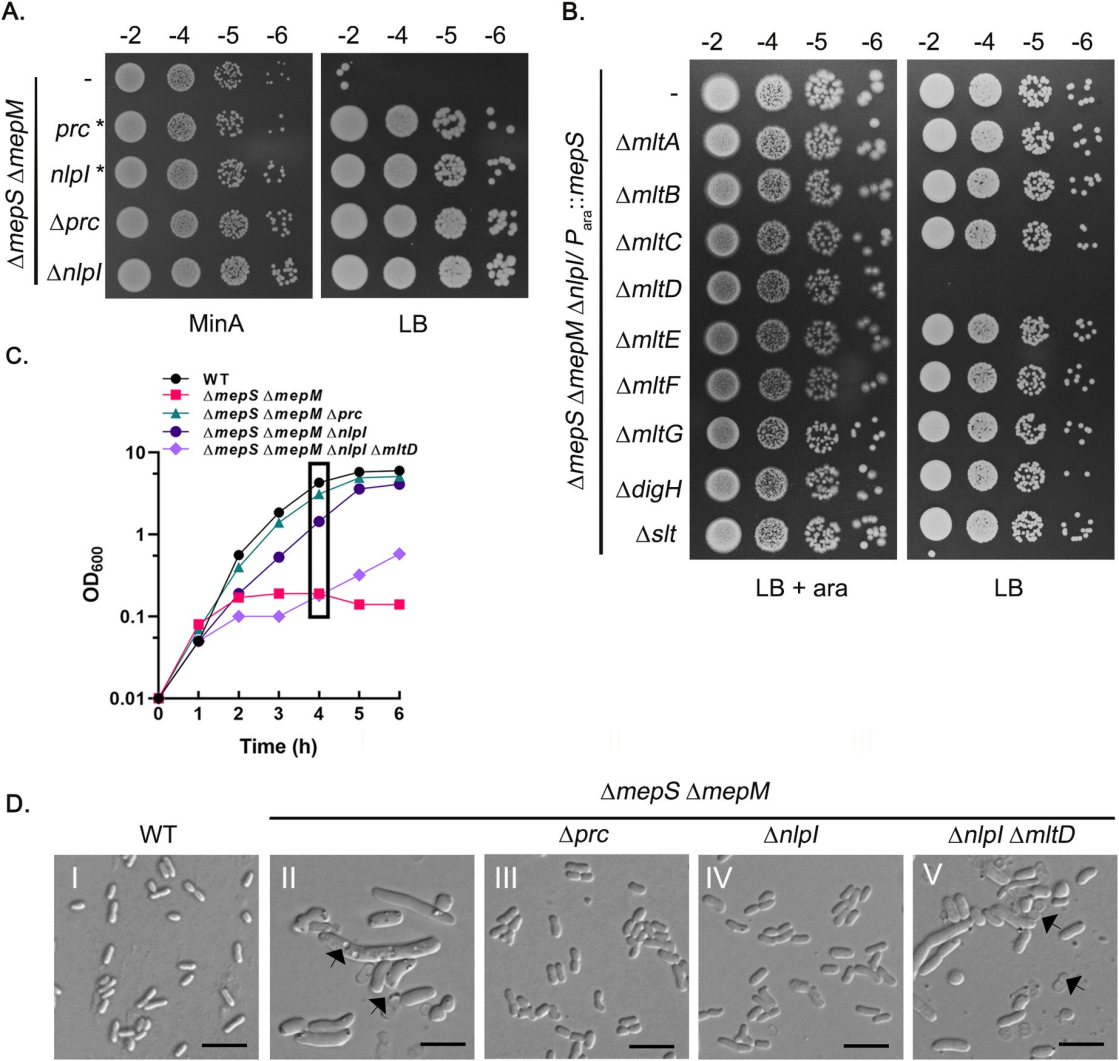
Absence of NlpI-Prc rescues the growth defects of *mepS mepM* mutant through MltD. (A) *mepS mepM* double mutant and its derivatives lacking either *nlpI* or *prc* were tested for viability on Minimal A medium (MinA) or LB plates. Cells were grown overnight in MinA, serially diluted and 4 μl of each dilution was spotted on indicated plates and grown overnight. prc* contains a 5 amino acid deletion in the signal sequence of *prc* gene whereas nlpI* contains a frameshift mutation at codon 21 (B) Δ*mepS* Δ*mepM* Δ*nlpI*/ P_ara::_*mepS* and its derivatives carrying deletion of each of the LTs were tested for viability on LB with and without arabinose (0.2%) (C,D) Cultures of WT (MG1655) and its mutant derivatives were grown overnight in MinA. Next day, they were washed and diluted 1:250 into fresh LB and growth was monitored at regular intervals. Cells were collected after 4 h of growth (marked) and visualized by DIC microscopy. Arrows indicate cell lysis. Scale bars represent 5 μm.

Absence of two major crosslink specific PG endopeptidases, MepS and MepM leads to rapid lysis of *E*. *coli* cells growing in rich media such as LB [6,11]. To understand the basis of cell lysis in these mutants, we sought to identify suppressor mutations that conferred viability to *mepS mepM* double deletion mutants on LB medium. Approximately 30 spontaneous suppressor mutants from two independently grown cultures were isolated and the mutations were identified using conventional gene mapping techniques (as described in Materials and Methods). Surprisingly, all these mutations were found to be recessive alleles of either *nlpI* or *prc* (Fig 1A). In support of this observation, the deletion alleles of *nlpI* or *prc* also suppressed the growth defects of *mepS mepM* double mutants on LB (Fig 1A). NlpI and Prc form a proteolytic complex in the periplasm in which NlpI functions as an adaptor protein to bring its substrate MepS to the proximity of Prc for its degradation [12,13]. The rescue of *mepS mepM* mutant by *nlpI* or *prc* gene deletions suggested the existence of an alternate PG endopeptidase that is stabilized in the absence of NlpI-Prc proteolytic machinery and thereby compensating the loss of MepS and MepM.

To identify the factor(s) responsible for the suppression, we deleted each of the known PG endopeptidases of *E*. *coli* (MepA, MepH, PBP4, PBP7 or AmpH) from *mepS mepM nlpI* triple deletion mutants; however, none of these deletions were able to abolish the growth of this triple mutant suggesting that the stabilization of these alternate endopeptidases is not the basis of the suppression (S1A Fig). In contrast to a recent observation which showed stabilization of MepH as the basis of suppression in Δ*mepS mepM* double mutant lacking NlpI or Prc [11], our results show that absence of NlpI-Prc system is still able to suppress the growth defect of *mepS*, -*M* and -*H* triple mutants (S1B Fig).

### MltD, an LTG mediates the suppression of ΔmepSM in absence of NlpI-Prc system

To identify the factor(s) potentially regulated by NlpI-Prc system, we took a candidate approach and introduced deletion of the genes encoding several PG hydrolases each involved in the synthesis, recycling, or turnover of PG into the *mepS mepM nlpI* triple mutant. Of all the deletions tested, we observed that deletion of *mltD*, a gene encoding a membrane-bound murein LTG was able to totally abrogate the growth of the triple mutant on LB (Fig 1B) suggesting that MltD could be the putative hydrolase responsible for the suppression of *mepS mepM* double mutant in the absence of NlpI. In support of this, the growth curves and microscopy (Fig 1C and 1D) showed that the absence of MltD leads to cell death and lysis in *mepS mepM nlpI* triple mutant. On the contrary, deletion of MltD in *mepS mepM prc* had only a minor effect on its viability suggesting Prc may have additional NlpI-independent substrates that function in PG expansion (S1C Fig). However, deletion of any other LTG encoded by *E*. *coli* did not affect the viability of *mepS mepM nlpI* triple mutant (Fig 1B).

### MltD is a substrate of NlpI-Prc proteolytic system

**Fig 2:**
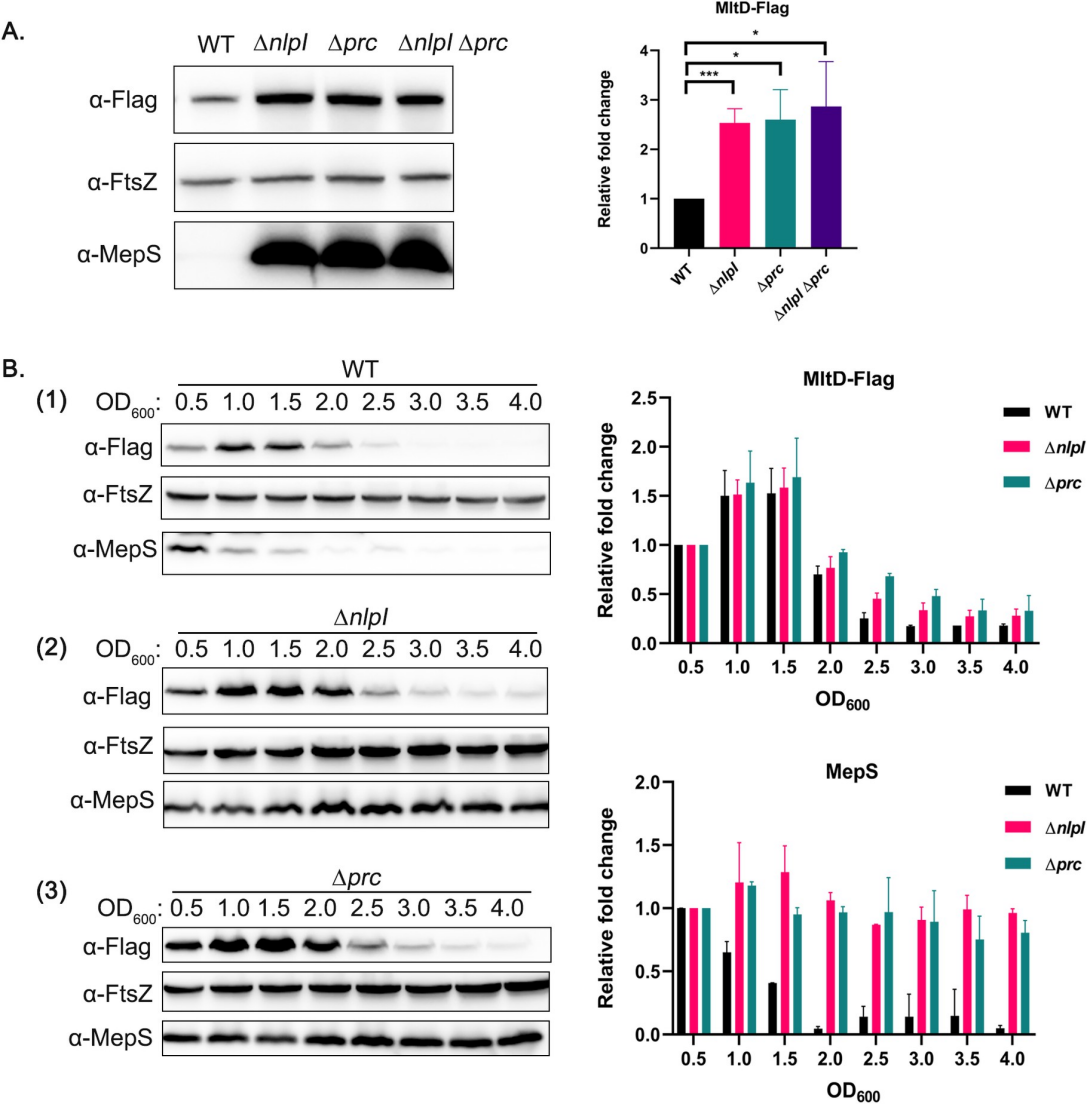
NlpI-Prc proteolytic system regulates MltD levels. (A) Indicated strains carrying *mltD*-Flag at their native chromosomal locus were grown in LB and cells were collected between OD_600_ of 0.8–1.0. Normalized cell fractions were subjected to SDS-PAGE followed by western blotting as described in Materials and Methods. MepS is used as a positive control. FtsZ is used as loading control to normalize the target protein. (B) Western blots showing the growth-phase specific expression of MltD-Flag in the indicated strains. Cells were grown in LB and fractions were collected at different OD_600_ values. Cell lysates were processed and analyzed as described above. Bar diagrams indicate the relative fold change of respective protein levels from three replicates; *, *P* <0.05; ***, *P* <0.001; ns (not significant); n = 3.

The above observations indicated that MltD is most likely regulated by NlpI-Prc system, prompting us to examine its level in strains lacking NlpI or Prc. For this, we constructed a functional C-terminal 3X Flag-tagged MltD at its native chromosomal locus (as described in SI) and examined its levels in WT, *nlpI*, *prc*, and *nlpI prc* mutants. Fig 2A shows that MltD level is approximately 2.5-fold higher in *nlpI* or *prc* single mutants and also in a *nlpI prc* double mutant compared to that of WT suggesting that NlpI and Prc together contribute to the maintenance of MltD. In addition, increasing the copy number of NlpI by introducing a plasmid-borne *nlpI* (P_ara_::*nlpI*) into a WT strain decreased MltD abundance (S2A Fig) confirming the ability of NlpI in modulating the level of cellular MltD. To examine whether other OM-associated LTGs are also regulated similarly by NlpI-Prc system, we checked the cellular levels of two LTGs, MltA (*mltA*-Flag) and MltF (*mltF*-Flag) in the absence of NlpI or Prc. Interestingly, MltF levels were approximately two-fold higher in absence of Prc but not in the absence of NlpI whereas MltA level is not affected by either of them (S2B Fig). It is known earlier that the levels of three other LTGs- MltB, MltG and DigH are modulated by Prc protease [22,27].

Furthermore, MltD level in WT *E*. *coli* strain was growth-phase specific; its levels were high in exponential phase of growth and gradually declined as cells entered stationary phase (Fig 2B). As expected, absence of NlpI or Prc elevated the MltD levels; however, interestingly, MltD still exhibited growth-phase specificity in these mutant backgrounds (Fig 2B). This observation is unlike that of MepS scenario wherein the growth-phase regulation of MepS is totally abolished in absence of functional NlpI-Prc system (Fig 2B).

**Fig 3:**
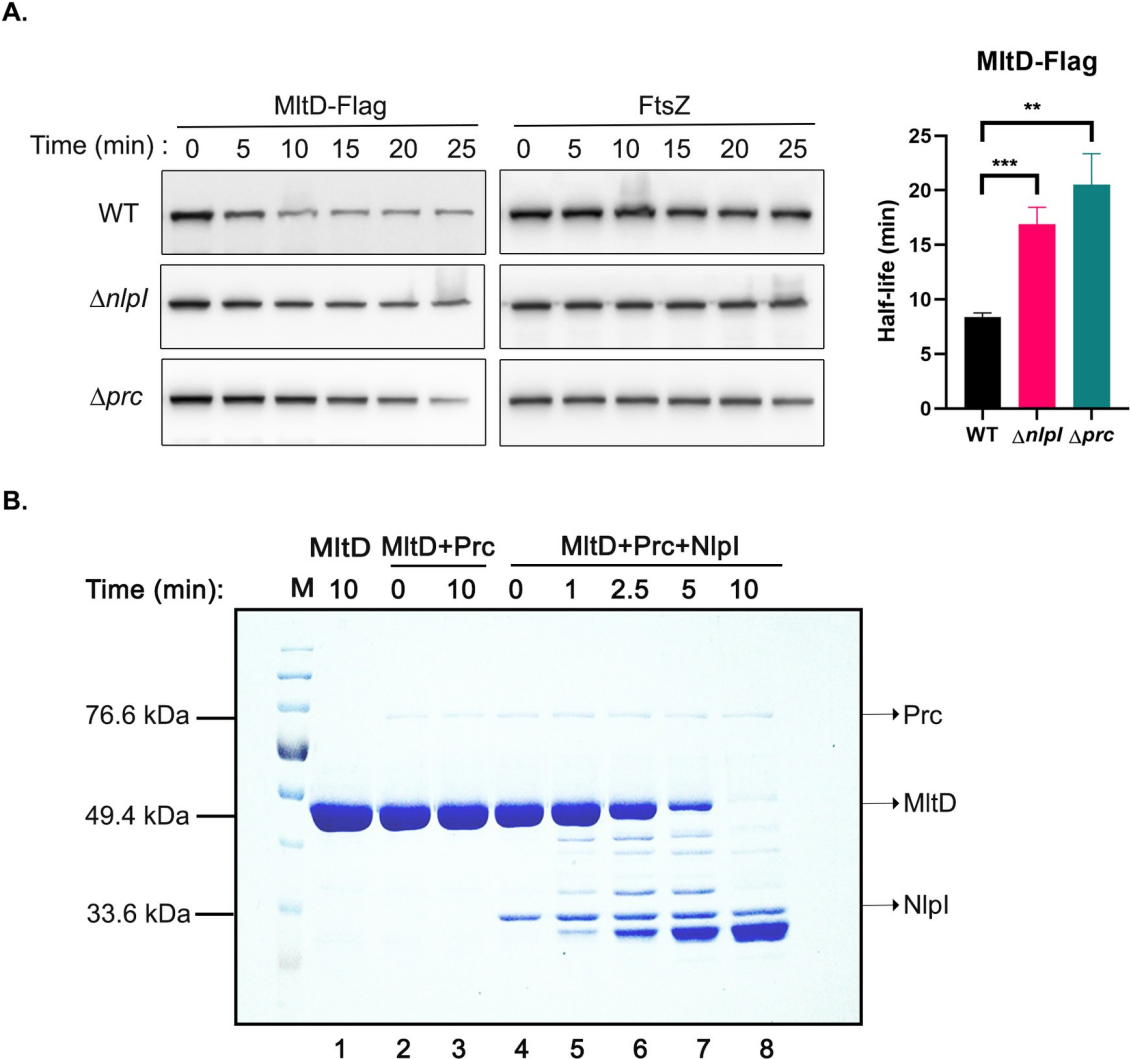
MltD is a substrate of NlpI-Prc proteolytic system. (A) Determination of half-life of MltD-Flag in the indicated strains was done as follows: cells were grown in LB till OD_600_ of 0.6 and 300 μg/ml of spectinomycin was added to block translation. Fractions were collected at indicated time points and were analyzed by western blotting as described in Materials and Methods. Error bars represent standard deviation. **, *P* <0.005; ***, *P* <0.001. FtsZ was used as a loading control. (B) *In vitro* degradation assay with purified MltD, Prc and NlpI proteins. The proteins were mixed in all combinations and incubated at 37°C followed by SDS-PAGE and Coomassie brilliant blue staining. Each reaction contained: MltD- 10 μg, NlpI- 1 μg and Prc- 0.4 μg. Molecular mass of the proteins is indicated in kDa.

As MepS and MepH levels are known to be controlled by NlpI-Prc at the step of post-translational stability [11,12], we speculated that MltD may also be similarly regulated. Hence, we measured the turnover of MltD in WT and its *nlpI* or *prc* derivatives by spectinomycin-chase experiments. Fig 3A shows that half-life of MltD in WT is approximately 8 min whereas in absence of NlpI-Prc, the half-life is increased up to 17–20 min indicating NlpI-Prc system partly contributes to MltD degradation.

### MltD is degraded by NlpI-Prc in vitro

Above results indicated that MltD is a substrate of NlpI-Prc proteolytic machinery *in vivo*. To test this observation *in vitro*, we overexpressed and purified MltD, NlpI and Prc lacking their signal sequences as hexahistidine fusion proteins (as described in SI). We then performed *in vitro* degradation assay by co-incubating these proteins and checked by SDS-PAGE. Fig 3B shows that MltD is rapidly degraded by Prc in the presence of NlpI (lanes 4–8) whereas co-incubation of MltD with Prc alone did not lead to its degradation even after prolonged incubation (lanes 2–3, Figs 3B and S3A). As MepS degradation is known to be similarly facilitated by NlpI-Prc [12], we tested whether MltD or MepS is the preferred substrate of NlpI-Prc proteolysis. Degradation assays done with MepS or MltD either individually or in combination indicated that both MltD and MepS are degraded by the NlpI-Prc system to a comparable extent (S3B Fig).

NlpI is known to bind MepS to facilitate its degradation by Prc [12,13]. To examine whether NlpI also physically interacts with MltD, we performed *in vivo* pull-down assays using a functional plasmid-borne NlpI-His (P_ara_::*nlpI-His*) as a bait (S3C Fig). However, here, NlpI-His did not show any interaction with MltD, although it was able to pull down MepS and Prc as shown earlier [12].

### MltD level is modulated by stationary-phase specific sigma factor, RpoS

**Fig 4:**
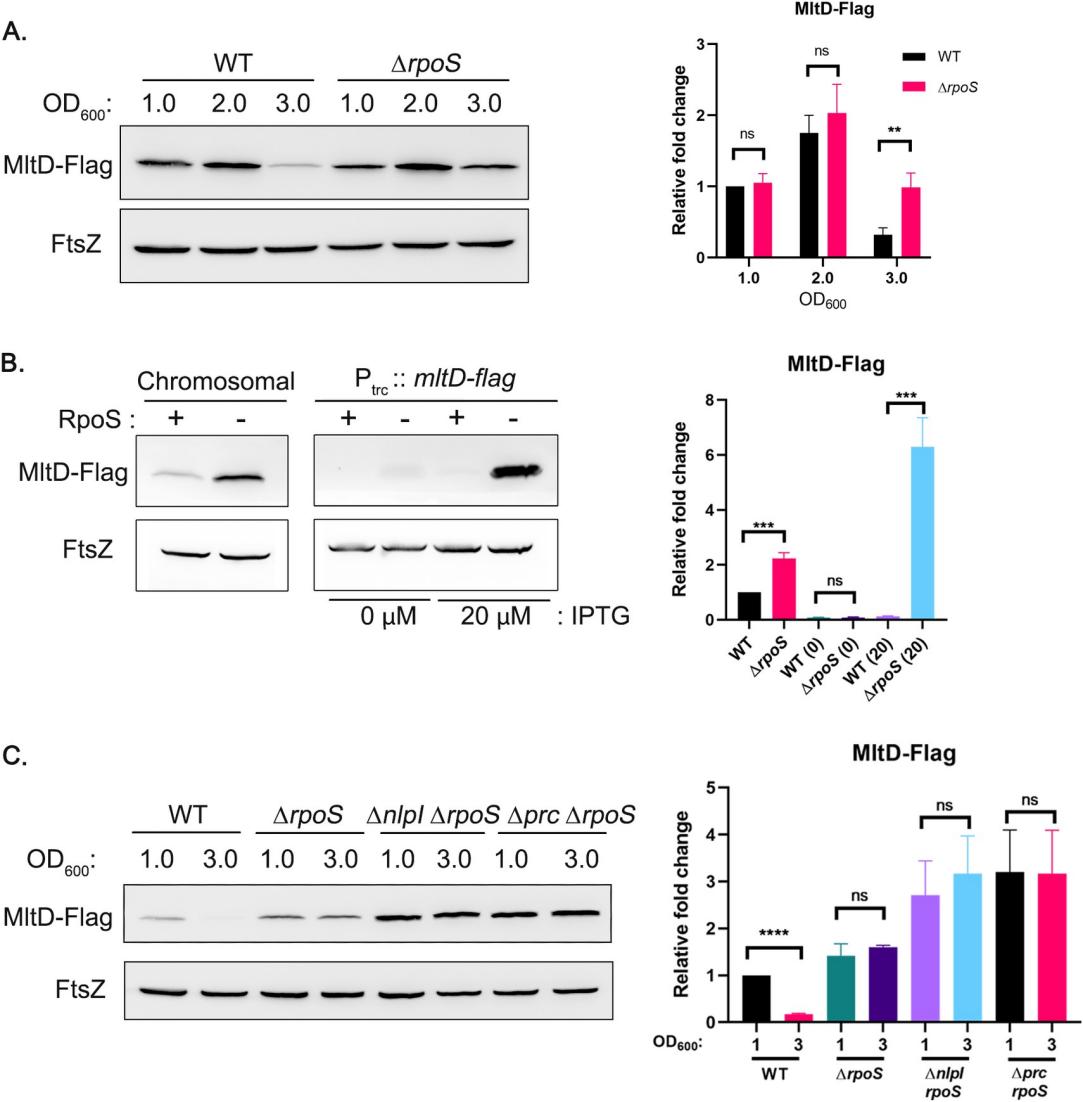
**Regulation of MltD by stationary-phase specific sigma factor, RpoS** (A) Indicated strains were grown in LB and fractions were collected at different OD_600_ values and MltD-Flag levels were analyzed by western blotting. (B) WT and Δ*rpoS* mutant strain carrying either chromosomal MltD-Flag or a plasmid-borne MltD-Flag (P_trc_::*mltD*-Flag) were grown in LB till OD_600_ of 3 and normalized cell lysates were subjected to SDS-PAGE followed by western blotting. (C) Indicated strains were grown in LB and cell fractions were collected at OD_600_ of 1.0 and 3.0. Normalized cell fractions were used for western blot analysis. Bar diagrams indicate the relative fold change of respective protein levels from three replicates; *, *P* <0.05; **, *P* <0.005; ***, *P* <0.001; ****, *P* <0.0001; ns (not significant); n = 3.

As the above data showed that MltD levels are lower in stationary-phase cells even in the absence of NlpI-Prc (Fig 2B), we hypothesized the existence of another layer of regulation that may operate to control the abundance of MltD in stationary-phase. To test this possibility, we examined the effect of a stationary-phase specific sigma factor, RpoS [28] on MltD levels. As speculated, absence of RpoS increased the level of MltD ~3-fold in the stationary-phase cells of *E*. *coli* (Fig 4A). RpoS is a subunit of RNA polymerase that influences transcription of several genes during growth of *E*. *coli* in various stress conditions including starvation, stationary-phase, and changes in osmolarity [28, 29]. To test the effect of RpoS on *mltD* gene expression, we constructed a chromosomal *lacZ* fusion downstream to the promoter of *mltD* (P_mltD_::*lacZY*; as described in SI) [30] and measured β-galactosidase levels. However, the *lacZ* levels remained identical in both WT and *rpoS* deletion strain suggesting that RpoS does not control *mltD* at the step of transcription (S4 Fig). To confirm this observation further, we constructed a plasmid-borne functional *mltD*-Flag (lacking its native promoter) downstream to an IPTG-inducible promoter (P_trc_::*mltD*-Flag) and examined MltD levels in the WT and *rpoS* mutant. Fig 4B shows that the plasmid-borne MltD level is highly elevated in the absence of RpoS showing that its control on MltD is promoter-independent and post-transcriptional. Further, the spectinomycin-chase experiments showed no significant change in the MltD degradation pattern in absence of RpoS compared to that of WT (S4B Fig), indicating that RpoS does not control MltD at the step of protein stability. Overall, these results show that RpoS does not regulate MltD at the step of transcription or post-translational stability but may indirectly influence MltD expression likely at the step of translation.

Next to check whether NlpI-Prc and RpoS act independently, we constructed double deletion mutants, *nlpI rpoS* and *prc rpoS* and examined the levels of MltD (Fig 4C). We find that in these strain backgrounds, the MltD levels remain highly stabilized all through the growth cycle indicating that NlpI-Prc and RpoS regulatory systems work in parallel to maintain the cellular levels of MltD.

### Overexpression of MltD confers growth to endopeptidase-deficient mutants

Because the above results indicated that MltD stabilization is the basis of Δ*mepSM* mutant suppression in absence of NlpI-Prc (Fig 1), we examined the effect of MltD overexpression on the growth of various endopeptidase-deficient mutants. We observed that a plasmid encoding *mltD* (cloned downstream of an IPTG-inducible promoter; ASKA collection) [31] confers considerable growth advantage to *mepS mepM* double mutant (S5A Fig). Interestingly, except *mltD*, overexpression of any other LTG encoded by *E*. *coli* (ASKA collection) did not confer growth to the Δ*mepSM* double mutant (S5B Fig). To confirm this, we cloned *mltD* downstream of an IPTG-inducible *trc* promoter in a medium-copy vector, pTrc99a (P_trc_::*mltD*) and this plasmid also conferred growth advantage to both *mepS* and *mepSM* double mutants (S5C and S5D Fig). To examine whether the enzymatic activity of MltD is obligatory for the suppression, we made catalytically inactive mutants of *mltD* (E125A and E125K) and observed that these inactive variants do not confer growth establishing the requirement of LTG activity for the suppression (S5C and S5D Fig). Further, a *mltD* derivative lacking the C-terminal LysM domains severely reduced the suppression suggesting that the interaction of MltD with PG is likely required for MltD activity (S5C Fig).

The rescue of endopeptidase-deficient mutants by MltD overexpression suggested a possibility of MltD activating other D,D-endopeptidases encoded by *E*. *coli* such as PBP4, PBP7, or MepA [9,10]. Hence, we constructed a *mepS* mutant lacking these three endopeptidases and observed that overexpression of MltD was still able to rescue the growth defect of this quadruple mutant (S5E Fig) indicating MltD does not work through any of these endopeptidases.

**Fig 5:**
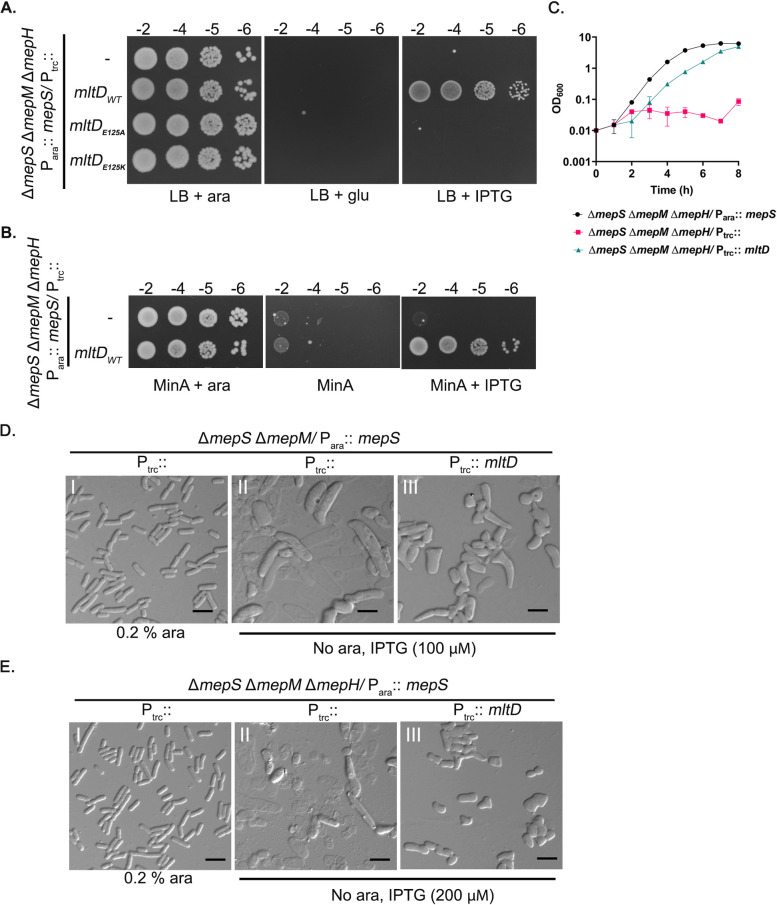
MltD overexpression restores growth to endopeptidase-deficient mutants. (A,B) Strains carrying pTrc99a or its *mltD* derivatives (WT, E125A, E125K) were grown overnight in LB with 0.2% arabinose and viability was checked on LB (A) or Minimal A (B) plates. IPTG was used at 250 μM (C) Strains were grown overnight with 0.2% arabinose and diluted 1:2500 into fresh LB containing appropriate inducers (0.2% arabinose or 250 μM IPTG) at 37°C and growth was monitored by OD_600_. (D, E) Cells were collected from cultures growing in LB (with arabinose or IPTG) after 3 h and subjected to DIC microscopy as described in Materials and Methods. Scale bars represent 5 μm. For panel II, cells from 20 ml culture were collected for microscopy.

However, notably, overexpression of *mltD* conferred considerable growth to a mutant lacking the three D,D-endopeptidases, MepS, -M and -H. *mepS mepM mepH* triple mutant is not viable on both LB and minimal synthetic media [6,11] and overexpression of MltD was able to rescue the lethality of this triple mutant in both LB and minimal medium (Fig 5A, 5B, and 5C). As MltD was able to compensate the lack of all the crucial crosslink cleaving endopeptidases of *E*. *coli*, we further investigated the suppression phenotype by examining the cell morphology of these mutants.

### Overexpression of MltD does not restore rod morphology to endopeptidase-deficient mutants

Viability assays and growth curve experiments showed that Δ*mepSMH* mutants grow reasonably well upon *mltD* overexpression (Fig 5A, 5B, and 5C). To check their morphology, we performed microscopic analysis of the cells grown with or without MltD overexpression. Panel II (Fig 5D and 5E) shows that the endopeptidase-deficient cells were extremely large and deformed with severe lysis. Overexpression of MltD in these cells (panel III, Fig 5D and 5E) decreased cell lysis, conferred cell viability, but interestingly, did not restore the cell shape. Most cells in the population although viable, were deformed with abnormal lateral wall protrusions, branching, and flattened poles indicating that MltD overexpression is sufficient to confer cell viability but not to restore an orderly rod-shaped structure to the PG sacculus.

### Deletion of MltD confers additive sickness to endopeptidase-deficient mutants

**Fig 6:**
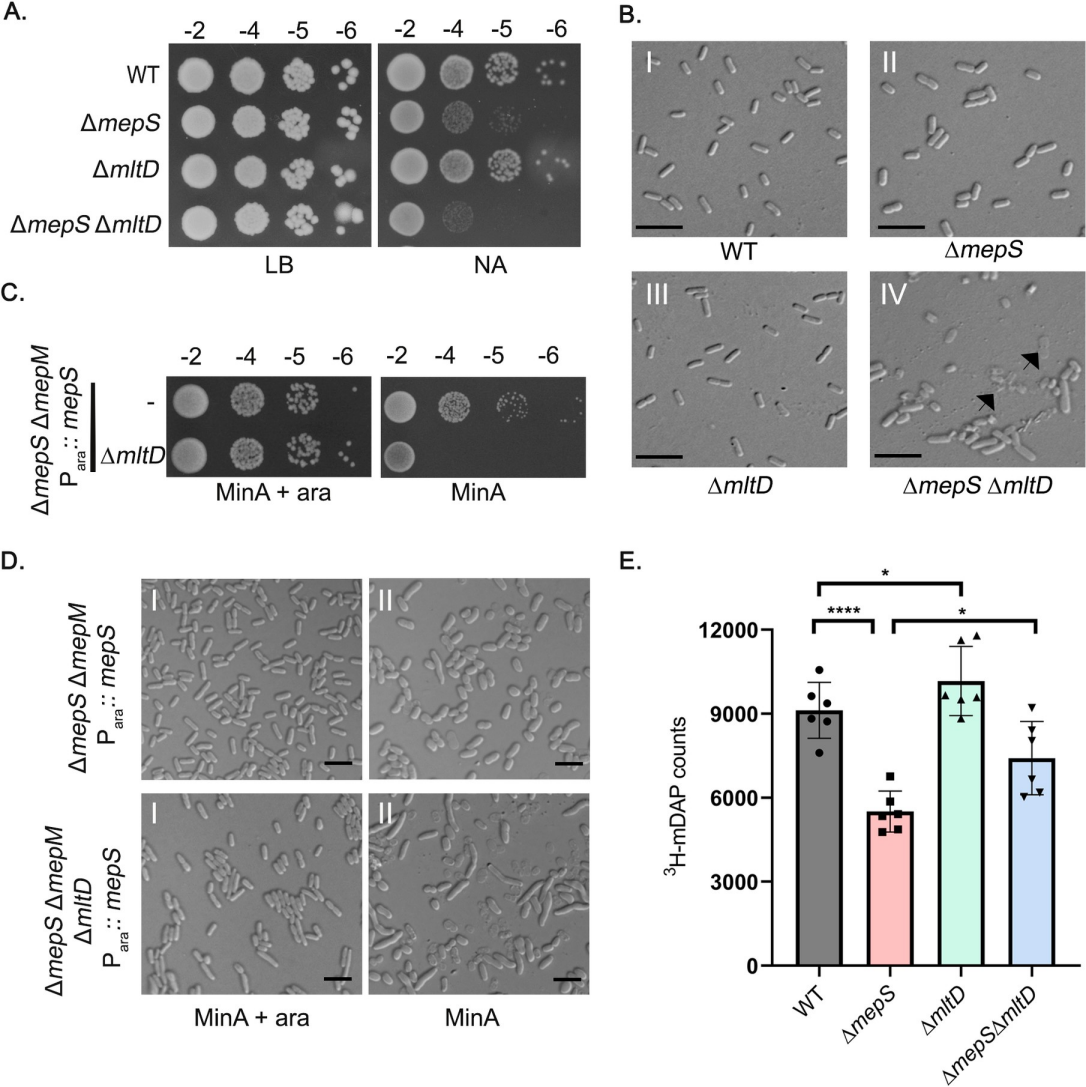
Effect of *mltD* deletion on endopeptidase-deficient mutants. (A) WT and its mutant derivatives were grown overnight in LB and viability assays were done on LB and NA plates at 30°C. (B) Overnight grown cultures of the above strains were washed and diluted 1:100 into fresh NB at 30°C and growth was monitored by OD_600_. Cells were collected after 5 h of growth and subjected to DIC microscopy as described in Materials and Methods. Arrows indicate lysed cells. (C) Indicated strains were grown in LB with 0.2% arabinose and viability was checked on indicated plates. (D) the cells were grown as described above in Minimal medium with 1:500 dilution for 10 h followed by DIC microscopy. Scale bars represent 5 μm. (E) Bar diagram depicting the incorporation of ^3^H-mDAP into the PG sacculi of WT and its mutant derivatives. Error bars represent standard deviation; *, *P* <0.05; ****, *P* <0.0001.

Because MltD overexpression rescued the lethality of the elongation-specific endopeptidase deletion mutants, we examined the effect *mltD* gene deletion in mutants lacking either *mepS* or *mepSM*. *mltD* deletion mutant by itself did not exhibit any discernible growth defect on LB, LBON or NA at any temperature or sensitivity to cell-wall antibiotics such as cefsulodin, mecillinam or vancomycin. However, deletion of *mltD* exacerbated the NA-sensitive phenotype of a *mepS* mutant (Fig 6A) and severely hampered the growth of *mepS mepM* double mutant on minimal media (Fig 6C). In addition, deletion of *mltD* significantly elevated cell lysis in *mepS* (Fig 6B) and *mepSM* mutants (Fig 6D) implying a role for MltD in PG expansion.

To further examine the role of MltD in PG synthesis, we measured the level of nascent PG strand incorporation using tritiated mDAP [32] in WT and its *mltD*, *mepS*, *mepS mltD* derivatives (Fig 6E). Surprisingly, the deletion of MltD itself showed a small, yet consistent increase in the mDAP incorporation (approximately 10% higher than the WT). Likewise, *mltD* deletion also increased the level of mDAP incorporation in *mepS* mutant background suggesting that these two PG hydrolases contribute differently to the expansion of PG.

### Absence of mrcA-lpoA partially restores rod shape to endopeptidase-deficient mutants expressing MltD

**Fig 7:**
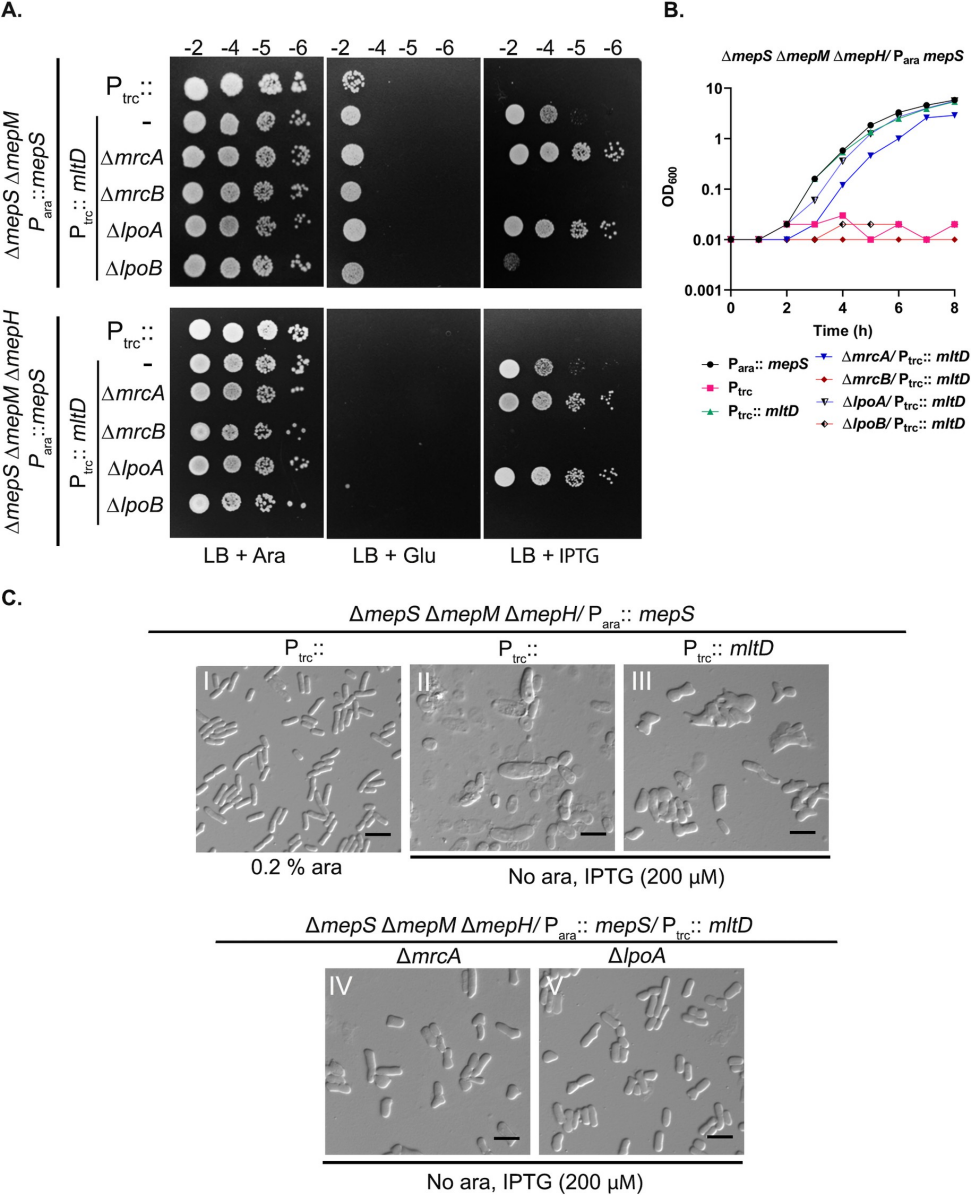
Absence of MrcA-LpoA restores growth to endopeptidase-deficient mutants overexpressing MltD. (A) Indicated strains were grown overnight in LB with 0.2% arabinose and viability was checked on plates. IPTG was used at 100 and 200 μM for top and bottom panel respectively. (B, C) Overnight grown cultures of the above strains were diluted 1:2500 into fresh LB containing appropriate inducers (0.2% arabinose or 200 μM IPTG) at 37°C and growth was monitored by OD_600_. Cells were collected after 3 h of growth and subjected to DIC microscopy as described in Materials and Methods. Scale bars represent 5 μm.

The lysis phenotype of endopeptidase-deficient cells and its suppression by MltD overexpression suggested a possibility that PG synthesis in absence of crosslink cleavage may uncouple glycan polymerization from crosslink formation leading to the generation of uncrosslinked, misincorporated glycan strands in the PG sacculi which are then cleaved by excess MltD. Therefore, we wondered whether lowering the wall synthesis by deleting any of the aPBPs (PBP1A encoded by *mrcA* or PBP1B by *mrcB*) or their cognate lipoprotein activators (LpoA or LpoB) may influence the growth of MltD-overexpressing endopeptidase-deficient mutants. To our surprise, we observed that deletion of *mrcA* considerably improved the growth of *mepSM* or *mepSMH* mutants expressing limited amount of MltD (Figs 7A, 7B, and S6A). Remarkably, the morphological aberrations were largely reduced with cells considerably regaining the rod morphology (Figs 7C and S6B). In contrast, deletion of *mrcB* completely abrogated the effect of MltD overexpression causing extreme sickness and lysis to these mutants. Deletions of *lpoA* or *lpoB* also behaved similar to that of *mrcA* or *mrcB* deletions confirming the above observations (Figs 7 and S6). Although preliminary, these results raised an interesting possibility of PBP1A-mediated PG synthesis being detrimental in the absence of endopeptidases. In absence of crosslink cleavage, PBP1A may generate glycan strands that remain poorly crosslinked with MltD likely functioning to remove these glycan strands for optimal and orderly expansion of PG.

### MltD cleaves uncrosslinked PG strands

**Fig 8:**
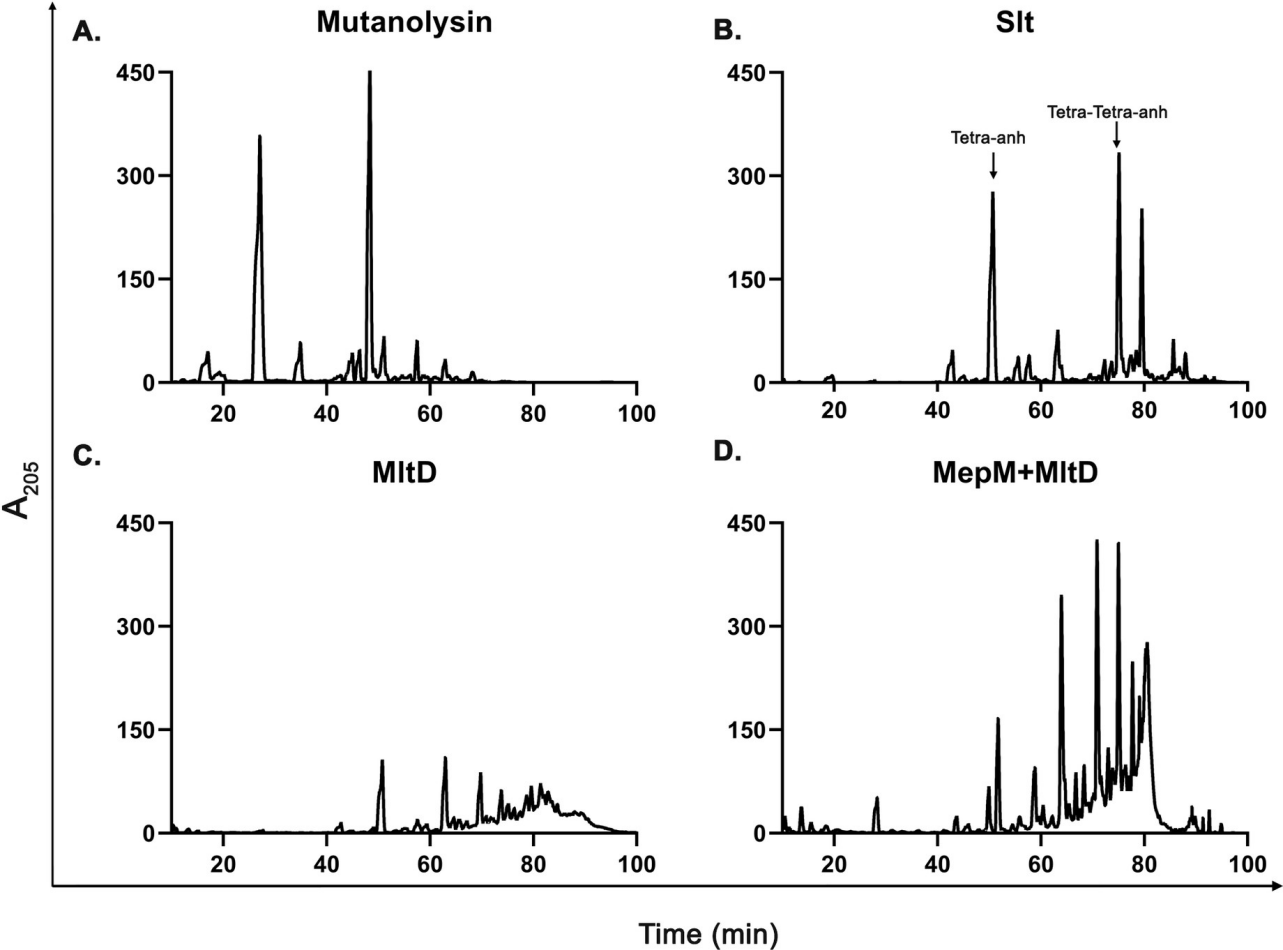
Enzymatic activity of MltD. HPLC chromatograms showing the activity of various muramidases (mutanolysin, Slt or MltD) on intact PG sacculi. Purified sacculi were treated with either mutanolysin, Slt or MltD (panel A, B, C) for 16 h at 37°C. (D) PG sacculi were treated with MepM for 16 h at 37°C followed by treatment with MltD. All proteins were used at 5 μM. *Tetra-anh (anhydro derivative of disaccharide tetrapeptide), Tetra-Tetra-anh (dimer of Tetra-anh).

Earlier studies suggested MltD may prefer uncrosslinked glycan strands as its substrate [18]; however, as the data is limiting, we attempted to further examine the substrate-specificity of MltD. For this purpose, we isolated PG sacculi from WT *E*. *coli* and treated them with purified MltD (Fig 8). After digestion, the soluble muropeptides were separated by RP-HPLC (reverse phase-high pressure liquid chromatography) and the chromatograms were analysed (Fig 8). The chromatograms show that the treatment of PG sacculi with MltD generates limited number of soluble muropeptides when compared to the treatment with other glycan cleaving enzymes such as mutanolysin (a commercially available muramidase) or Slt, a soluble periplasmic LTG. To check whether MltD prefers uncrosslinked glycans, we treated PG sacculi with MepM, a 4,3-crosslink specific D,D-endopeptidase [6]. These MepM-treated PG sacculi were then used for digestion with MltD and the chromatograms show that MltD is able to hydrolyse the uncrosslinked PG sacculi with higher efficiency compared to that of intact PG sacculi (Fig 8C vs 8D) indicating that MltD prefers uncrosslinked glycan strands as its substrate.

## Discussion

In this study, we show a previously unidentified role for a LTG, MltD in PG expansion of *E*. *coli*. PG expansion requires the cleavage of crosslinks to open the mesh for generating space to incorporate new PG material. In *E*. *coli*, the ‘space-making activity’ is collectively achieved by MepS, -M and -H, whose absence leads to rapid cell lysis. Here, we observed that overexpression of MltD that cuts the glycan polymers of the PG sacculus rescues the cell lysis caused by the absence of crosslink cleavage. Further detailed genetic analysis suggested that the LTG activity of MltD contributes to PG expansion by alleviating the deleterious defects arising due to PG synthesis during endopeptidase insufficiency. We also find that MltD levels are stringently controlled by two independent regulatory pathways; one at the step of post-translational stability by a periplasmic adaptor-protease complex, NlpI-Prc, and another, by RpoS, a stationary-phase specific sigma factor highlighting the need for the maintenance of optimal level of MltD during growth of *E*. *coli*.

### Role of MltD in PG expansion

MltD is one of several membrane-bound LTGs encoded by *E*. *coli* [10,18,33]. It is an OM lipoprotein containing an N-terminal transglycosylase domain and two LysM (lysin) repeats which facilitate PG binding (S5F Fig) [26]. It is reported to be an endolytic LT that may prefer uncrosslinked substrates; however, its role in PG metabolism was not clear [17]. Our initial finding of MltD stabilization as the basis of suppression of *mepSMH* mutant in absence of NlpI-Prc indicated a potential role for MltD in PG expansion (Fig 1). Notably, overexpression of MltD compensating the absence of all the three essential crosslink-specific endopeptidases signified the importance of glycan cleavage in PG expansion. The cell morphology of *mepSM*/ *mepSMH* deletion mutants and their derivatives expressing MltD was instrumental in hypothesizing the function of MltD in PG expansion (Fig 5). MltD overexpression restoring viability, but not rod morphology, to the endopeptidase-defective mutants implied that MltD activity may differ from the canonical space-making function attributed to the crosslink cleaving enzymes during PG incorporation [8]. Remarkably, removal of PBP1A or LpoA conferred rod morphology to MltD-overexpressing endopeptidase-deficient cells (Figs 7 and S6), raising the possibility that absence of crosslink cleavage leads to the lethal accumulation of uncrosslinked glycan polymers by PBP1A-LpoA, which are removed by the LTG activity of MltD. Consistent with this model, *mltD* deletion confers an increase in the rate of mDAP incorporation (Fig 6E), likely due to the nascent glycan strands being retained in the PG sacculus in the absence of MltD. Although the above findings support a quality control function for MltD in processing the uncrosslinked glycan strands generated by PG synthases; however, at this stage, we cannot rule out an additional role for MltD in cleaving the glycans of the mature PG matrix to make space during its expansion.

Although *E*. *coli* encodes several LTGs reflecting their importance in various cellular processes [17,19,25], a mutant lacking multiple LTGs has no major phenotype, hindering the progress in understanding their specific functions. Recent studies have revealed distinct roles for few LTGs in various PG pathways [23,24,33]. For example, MltG determines the glycan chain length during PG polymerization [21]; and DigH cleaves denuded glycan strands at the cell septum for cell separation [22]. In addition, recent studies in *V*. *cholerae* show that LTGs have an essential role in mitigation of periplasmic crowding due to soluble PG turnover products [24]. Most importantly, Slt removes uncrosslinked nascent glycan strands generated due to the inhibition of crosslink formation during scaffold synthesis by the Rod complex in *E*. *coli* [23]. Along similar lines, we speculate that MltD may work towards removing the uncrosslinked polymers generated during sidewall synthesis. Moreover, these results also indicate that PBP1A but not PBP1B, preferentially generates uncrosslinked polymers in the absence of crosslink cleavage, raising several questions regarding the differential role of the aPBPs in PG expansion. To summarize, our results provide evidence for MltD in removing the uncrosslinked glycan material generated during PG expansion to provide orderly insertion of new strands for proper cell shape and cell wall integrity.

At this juncture, it is not clear why MltD is unique among the *E*. *coli* LTGs in its ability to rescue the endopeptidase mutants when overproduced. It could be due to its OM-localization, its endolytic muramidase activity, its preference for uncrosslinked glycan strands or a combination of all these factors.

### Regulation of MltD

Although PG hydrolases are fundamental for various cell wall processes, they need to be tightly regulated as their unfettered activity might lead to PG degradation with loss of envelope integrity. In support of this, we find MltD levels are controlled by two independent pathways. Both *in vivo* and *in vitro* experiments confirmed MltD to be a substrate of NlpI-Prc system (Fig 3A and 3B). A recent study also reported the stabilization of MltD in absence of NlpI-Prc proteolytic complex in *E*. *coli* [34]. Till date, MepS [12] and MepH [11], the crosslink specific endopeptidases involved in PG enlargement are the only substrates identified to be dependent on NlpI for their turnover by Prc. In contrast, Prc protease alters the stability of several periplasmic PG enzymes in NlpI-independent manner [16,27]. The fact that three PG hydrolytic enzymes required for PG expansion, MepS, MepH and MltD are controlled by NlpI-Prc system raises an interesting possibility of NlpI playing a larger role in regulating the process of PG expansion by modulating the stability of these hydrolases. Interestingly, the turnover of both MepS and MltD is controlled by the NlpI-Prc system, suggesting the existence of a shared regulatory pathway that responds to the cellular need of PG expansion. A recent study shows that cellular fatty acid or phospholipid availability modulates the stability of MepS and MltD through NlpI-Prc to coordinate the cell envelope biogenesis [35]. During cell elongation, their stabilization may lead to successful PG expansion to achieve orderly insertion of new PG strands; and when PG synthesis is not needed, MepS and MltD may undergo NlpI-Prc dependent proteolysis thus avoiding a futile round of PG degradation.

In addition to NlpI-Prc control which may operate during cell wall expansion, MltD levels are kept in check in stationary-phase by RpoS, the stationary-phase specific sigma factor (Fig 4). Our results show that the regulation by RpoS is neither at the level of transcription nor at the level of protein stability. It would be interesting to examine how RpoS exerts its control on MltD expression.

## Materials and methods

### Media and growth conditions

Strains were normally grown in Luria-Bertani (LB) (1% tryptone, 0.5% yeast extract and 1% NaCl) broth. LBON is LB without NaCl. Nutrient Agar (NA) had 0.5% peptone, 0.3% beef extract and 1.5% agar. Minimal-A medium [36] was supplemented with B1, MgS0_4_ and 0.2% glucose before use. Casamino acids (CAA) were used at 0.5%. Antibiotics were used as following: kanamycin (Kan)- 50 μg/ml; chloramphenicol (Cm)- 25 μg/ml; ampicillin (Amp)- 50 μg/ml. Isopropyl β-D-thiogalactopyranoside (IPTG) and L-arabinose were used at the indicated concentration. Growth temperature was 37°C unless otherwise specified. Growth was monitored by measuring optical density at 600nm (OD_600_).

### Molecular and genetic techniques

Recombinant DNA techniques, P1-phage mediated transductions and transformations were performed using standard methods [36]. Deletion mutations were sourced from Keio mutant collection [37]. Whenever required, the kanamycin resistance gene was flipped out using pCP20 plasmid encoding FLP recombinase [38]. The 3X Flag epitope tagging was done at the C-terminus of the ORF at its native chromosomal locus by recombineering [39].

### Viability assays and microscopy

Viability of strains was tested by serially diluting (10^−2^, 10^−4^, 10^−5^ and 10^−6^) the overnight-grown cultures and applying 4 μl of each dilution onto the indicated plates followed by incubation for 18–36 h at the indicated temperature. Growth was recorded by photographing the plates. For microscopic analysis, overnight cultures were subcultured and grown till the required OD. Cells were collected, immobilized onto a thin 1% agarose pad and visualized using a Zeiss apotome microscope by DIC (Nomarski optics).

### Plasmid constructions

For PCR amplifications, genomic DNA of MG1655 strain was used as a template. Amplification of DNA was done using Q5 High-Fidelity DNA polymerase (NEB) and clones obtained were confirmed by sequence analysis. Plasmid constructions are described in detail in SI.

### Screen for isolation of spontaneous suppressors of *mepS mepM* double mutant

A strain carrying deletion of both *mepS* and *mepM* was grown overnight in minimal-A broth and the next day, approximately 10^8^ cells were plated on LB agar and incubated at 37°C. Approximately 30 colonies that were able to grow on LB plates were picked up and purified. The suppressors were broadly classified based on the extent of suppression and a few of these were initially mapped using conventional conjugational and transductional mapping techniques [36]. Interestingly, these suppressor mutations turned out to be recessive alleles of either *nlpI* or *prc*; subsequently we tested all the remaining suppressors and to our surprise all the short-listed suppressors were mapped to either *nlpI* or *prc*.

### Western blotting

For collection of samples for the western blot analysis, overnight cultures were subcultured and grown till indicated OD_600_ value. Growth was carefully monitored by measuring the culture density (OD_600_) at regular intervals and cells were collected. Normalized cell fractions were immediately pelleted at 4°C, resuspended in 1X Laemmli loading buffer and boiled for 10 min. Samples were centrifuged before being resolved using SDS-PAGE (14%). Proteins were transferred onto a PVDF membrane using semi-dry western transfer method. Bands were visualized by Ponceau S stain to ascertain protein transfer. Membrane was blocked by 5% skimmed milk in 1X TBST for 1 h and then incubated overnight with primary antibodies [1:3,000 for α-MepS (kind gift from Waldemar Vollmer), 1:5,000 for α-NlpI (laboratory collection), 1:4,000 for α-Flag (F3165, Sigma-Aldrich, USA), 1:10,000 for α-FtsZ (kind gift from Joe Lutkenhaus), at 4°C under constant shaking. The membrane was then washed four times with 1 X TBST (Tris-buffered saline with 0.01% Tween-20) for 5 min each and then probed with secondary antibodies (dilution of 1:10,000) tagged to HRP (horseradish peroxidase) enzyme and further incubated for 1 h. Membrane was then washed with 1X TBST four times for 5 min each to remove unbound antibodies. Proteins were visualized using enhanced chemiluminescence (ECL)-prime detection substrate (Amersham) inside a chemi-documentation system. Bands were quantitated using ImageJ software. FtsZ is used as loading control to normalize the target protein. Fold-change was calculated after normalization with the FtsZ values. All the experiments were done three times and the representative blots are shown.

### Half-life determination

To examine the MltD protein degradation *in vivo*, spectinomycin-chase experiments were performed. To rapidly growing cells in LB (at the required OD), spectinomycin (300 μg/ml) was added to inhibit the protein synthesis and cells were collected at indicated time points. Normalized cell fractions were processed by western blotting as described above.

### mDAP incorporation assay

Incorporation of ^3^H-mDAP (tritiated mDAP) into the PG sacculi was done as described [32]. Indicated strains (lacking LysA to block the formation of lysine from mDAP) were grown overnight in LB broth, and the next morning, they were washed, and diluted 1:100 in prewarmed minimal-A medium containing 0.5% CAA as a carbon source. At an OD of 0.5–0.6, normalized cell fractions were collected and incubated with 5 μCi/ml of ^3^H-mDAP (^3^H- DAP;14.6 Ci mmol-1, Moravek Biochemicals, USA) for 10 min with gentle shaking at 37°C. Cells were immediately lysed by addition of 3 ml of 4% SDS and boiled for 1 h. The mixture was cooled overnight at RT and filtered through 0.22 μm filter (Millipore). The insoluble PG sacculi were collected on the filters, washed with 30 ml of Milli-Q water and the filters were dried completely before counting the radioactivity in a liquid scintillation counter (Perkin-Elmer).

### Protein overexpression and purification

MltD, NlpI, Prc, MepS and MepM proteins (lacking their periplasmic signal sequences) fused to hexahistidine tags were overexpressed and purified to homogeneity from BL21 (λDE3) or its derivatives as described in detail in SI. MltD and MepS had N-terminal His tag whereas NlpI, Prc, and MepM were fused to C-terminal His tag. Proteins were purified through metal-affinity chromatography using Ni^2+^–NTA agarose beads (Qiagen). Purified protein aliquots were stored at -30°C in 50 mM Tris-Cl, 100 mM NaCl, 1mM DTT and 50% glycerol for further use.

### *In vitro* degradation assay

The indicated purified proteins were mixed and incubated at 37°C for different time intervals as described earlier [12]. Reactions were stopped by addition of 4X Laemmli buffer and boiling for 10 min. Samples were separated by SDS-PAGE followed by staining with Coomassie brilliant blue. Each reaction contained 10 μg MltD, 1 μg NlpI and 0.4 μg Prc. Proteins were added and mixed in the reaction buffer (20 mM Tris-Cl pH 8.0, 150 mM NaCl) on ice.

### Isolation of PG sacculi

PG sacculi were isolated as described earlier with slight modifications [2,6]. In brief, cells from 1 L of exponentially growing culture of WT (MG1655) were harvested by centrifugation followed by resuspension of the cell pellet in 9 ml of ice-cold deionised water. The cell suspension was added drop wise to 9 ml of boiling 8% SDS with continuous stirring and further boiled for 60 min. This mixture was stored overnight at RT and the sacculi were collected by ultracentrifugation (400,000 g for 60 min at RT). The pellet was washed repeatedly with deionised water to remove SDS. The high molecular weight glycogen and covalently bound lipoprotein were removed by adding α-amylase (100 μg/ml in 10 mM Tris-HCl, pH 7.2 for 2 h at 37°C) and heat-activated pronase (200 μg/ml for 90 min at 60°C) respectively. Samples were boiled with equal volume of 8% SDS for 15 min to inactivate these enzymes. Sacculi were collected by ultracentrifugation and washed repeatedly with deionized water to remove traces of SDS. Finally, the pellet was resuspended in 25 mM Tris-HCl (pH 8.0) and stored at -30°C.

### PG analysis by RP-HPLC

HPLC analysis was performed as described earlier [2,6] with some modifications. PG sacculi were treated with appropriate enzymes for 16h at 37°C, the proteins were heat-inactivated through boiling, and insoluble material was removed by centrifugation (14,000 RPM at RT). Subsequently, the soluble muropeptides were collected and mixed with equal volume of 50 mM sodium borate buffer (pH 9.0). The anomeric carbons of muropeptides were reduced by adding 1 mg of sodium borohydride. Excess borohydride was destroyed by adding 1/20^th^ volume of orthophosphoric acid and pH was adjusted between 2–4 before loading onto a Zorbax 300 SB RP-C18 (250 X 4.6mm, 5 μm) column. Separation of muropeptides was done by RP-HPLC using Agilent technologies RRLC 1200 system. Samples were injected onto a preheated column at 55°C and binding was allowed at a flow rate of 0.5 ml per minute with a solvent containing 1% acetonitrile and 0.1% trifluoroacetic acid (TFA) for 10 min. A gradient of 1–10% (MepM treated substrates) or 1–15% (untreated PG) acetonitrile containing 0.1% TFA was used for final elution at a flow rate of 0.5 ml per minute. Absorbance was detected at 205 nm.

### Statistical analysis

The experiments were conducted a minimum of three times, and statistical significance was determined based on the obtained data. The unpaired Student t-test was employed to calculate the statistical significance between two groups, with a confidence value of 0.05 considered significant for all tests. In the figures presented, asterisks (*) denote p-values less than 0.05, double asterisks (**) indicate p-values less than 0.01, triple asterisks (***) represent p-values less than 0.001 and quadruple asterisks (****). Error bars in the graphs are shown as mean ± SD. Data plotting and analysis were performed using GraphPad Prism 8.0.

## Supporting information

S1 Text Details of strain and plasmid constructions, supplemental methods, and protocols are described. **Table A.** List of strains used in this study. **Table B.** List of plasmids used in this study.
(DOCX)

S1 Fig Absence of NlpI-Prc system rescues the growth defects of *mepS mepM* mutant through MltD. (A) Indicated strains carrying deletion of each of the known D,D-endopeptidases were tested for viability on LB with and without arabinose (0.2%). (B) Indicated strains and its mutant derivatives lacking *nlpI* or *prc* were tested for viability on LB with and without arabinose (0.2%). (C) Δ*mepSM* double mutant or its derivatives were grown overnight in MinA and their viability was tested on MinA, LBON (LB without NaCl) or LB plates.
(TIF)

S2 Fig Regulation of LTGs by NlpI-Prc system. (A) Western blot showing the level of MltD-Flag in indicated strains. Cells were grown in LB supplemented with arabinose and fractions were collected between OD_600_ of 0.8–1.0. Normalized cell fractions were subjected to SDS-PAGE and analyzed by western blot. (B) WT and its mutant derivatives carrying *mltA*-Flag or *mltF*-Flag at their native chromosomal locus were grown in LB and processed as described above. Bar diagrams indicate the relative fold change of respective protein levels from three replicates; *, *P* <0.05; **, *P* <0.005; ****, *P* <0.0001; ns (not significant); n = 3.
(TIF)

S3 Fig Interaction between MltD, NlpI and Prc proteins. (A) *In vitro* degradation assay with purified MltD, Prc and NlpI proteins. Proteins were mixed in different combinations and incubated at 37°C followed by SDS-PAGE. Amounts of proteins used are as follows: MltD- 10 μg, NlpI- 1 μg, Prc- 0.4 μg. (B) *In vitro* degradation assay with purified MepS, MltD, Prc and NlpI proteins. Left panel shows degradation of MepS or MltD. Right panel shows the degradation of both MepS and MltD. (C) *In vivo* pull-down assay to check the interaction of NlpI with MltD. Plasmid borne NlpI-His was used as a bait to examine its interaction with MltD-Flag as described in SI. Prc-HA and MepS are used as positive controls.
(TIF)

S4 Fig Regulation of MltD by stationary-phase specific sigma factor, RpoS. (A) β-galactosidase values of P_*mltD*_:: *lacZY* in WT and *rpoS* deletion mutant at OD_600_ of 0.6 and 3.0. Assays were performed as described in SI. Values are calculated as Miller units and indicated as bar graphs. (B) Half-life of MltD-Flag in the indicated strains was checked as follows: cells were grown in LB till OD_600_ of 0.6 or 3.0 and 300 μg/ml or 1 mg/ml of spectinomycin was added to block translation. Fractions were collected at indicated time points and were analysed by western blotting, as described in Materials and Methods. Error bars represent standard deviation. ns–not significant. FtsZ was used as a loading control.
(TIF)

S5 Fig Overexpression of MltD restores viability to endopeptidase-deficient mutants. (A) Cells of *mepS mepM* mutant carrying pCA24N (ASKA empty vector) or pCA24N-*mltD* were grown overnight in MinA medium and viability was tested on LB plates with IPTG (25 μM). (B) Growth of indicated strains was checked on MinA or LB plates with IPTG (20 μM). pCA24N-MltB clone was not used in this experiment. (C, D, E) Indicated strains carrying pTrc99, pTrc99a-*mltD* or its derivatives were grown overnight in LB and viability was tested on indicated plates. NA plates had 10 μM IPTG whereas LB plates had 100 μM IPTG. (F) Depiction of structural features of MltD protein indicating its transglycosylase domain and two C-terminal LysM repeats.
(TIF)

S6 Fig Effect of aPBP deletions in MltD-overexpressing endopeptidase-deficient mutants. (A, B) Overnight grown cultures of the indicated strains were diluted 1:2500 into fresh LB containing appropriate inducers (0.2% arabinose or 100 μM IPTG) at 37°C and growth was monitored by OD_600_. Cells were collected after 3 h of growth and subjected to DIC microscopy as described in Materials and Methods. Scale bars represent 5 μm.
(TIF)

## References

[1] Höltje J-V (1998) Growth of the stress-bearing and shape-maintaining murein sacculus of *Escherichia coli*. Microbiol Mol Biol Rev 62: 181–203. 9529891 10.1128/mmbr.62.1.181-203.1998 PMC98910

[2] Glauner B, Holtje JV, Schwarz U (1988) The composition of the murein of *Escherichia coli*. J Biol Chem 263: 10088–10095. 3292521

[3] Egan AJF, Errington J, Vollmer W (2020) Regulation of peptidoglycan synthesis and remodelling. Nat Rev Microbiol 18: 446–460. doi: 10.1038/s41579-020-0366-3 32424210

[4] Garde S, Chodisetti PK, Reddy M (2021) Peptidoglycan: structure, synthesis, and regulation. EcoSal Plus 9: 629–646. doi: 10.1128/ecosalplus.ESP-0010-2020 33470191 PMC11168573

[5] Tomasz A (1984) Building and breaking of bonds in the cell wall of bacteria- the role for autolysins. Microbial cell wall synthesis and autolysis. Elsevier pp.3–12.

[6] Singh SK, Saisree L, Amrutha RN, Reddy M (2012) Three redundant murein endopeptidases catalyse an essential cleavage step in peptidoglycan synthesis of *Escherichia coli* K12. Mol Microbiol 86:1036–1051. 23062283 10.1111/mmi.12058

[7] Dörr T, Cava F, Lam H, Davis BM, Waldor MK (2013) Substrate specificity of an elongation-specific peptidoglycan endopeptidase and its implications for cell wall architecture and growth of *Vibrio cholerae*. Mol Microbiol 89:949–962. 23834664 10.1111/mmi.12323 PMC3769093

[8] Rajguru V, Chatterjee S, Garde S, Reddy M (2023) Crosslink cleaving enzymes: the smart autolysins that remodel the bacterial cell wall. Trends Microbiol. S0966842X23003220. doi: 10.1016/j.tim.2023.11.004 38072724

[9] Vollmer W, Joris B, Charlier P, Foster S (2008) Bacterial peptidoglycan (murein) hydrolases. FEMS Microbiol Reviews 32:259–286. doi: 10.1111/j.1574-6976.2007.00099.x 18266855

[10] Heijenoort JV (2011) Peptidoglycan hydrolases of *Escherichia coli*. Microbiol Mol Biol Rev. 75: 636–663. 22126997 10.1128/MMBR.00022-11 PMC3232740

[11] Jeon WJ, Cho H (2022) A cell wall hydrolase MepH is negatively regulated by proteolysis involving Prc and NlpI in *Escherichia coli*. Front Microbiol 13: 878049. 35418955 10.3389/fmicb.2022.878049 PMC8996183

[12] Singh SK, Parveen S, SaiSree L, Reddy M (2015) Regulated proteolysis of a cross-link-specific peptidoglycan hydrolase contributes to bacterial morphogenesis. Proc Natl Acad Sci USA 112:10956–10961. doi: 10.1073/pnas.1507760112 26283368 PMC4568209

[13] Su MY, Som N, Wu CY, Su SC, Kuo YT, Ke LC et al (2017) Structural basis of adaptor-mediated protein degradation by the tail-specific PDZ-protease Prc. Nat Comm 8: 1–13. doi: 10.1038/s41467-017-01697-9 29138488 PMC5686067

[14] Srivastava D, Seo J, Rimal B, Kim SJ, Zhen S, Darwin AJ (2018) A proteolytic complex targets multiple cell wall hydrolases in Pseudomonas. mBio 9e00972-18. 10.1128/mBio.00972-18 PMC6050968 30018106

[15] Chakraborty D, Darwin AJ (2021). Direct and indirect interactions promote complexes of the lipoprotein LbcA, the CtpA protease and its substrates, and other cell wall proteins in *Pseudomonas aeruginosa*. J Bacteriol 203: 003932. 10.1128/JB.00393-21 PMC8604077 34570626

[16] Sommerfield AG, Darwin AJ (2022) Bacterial carboxyl-terminal processing proteases play critical roles in the cell envelope and beyond. J Bacteriol 204: e00628–21. doi: 10.1128/jb.00628-21 35293777 PMC9017358

[17] Dik DA, Marous DR, Fisher JF, Mobashery S (2017) Lytic transglycosylases: concinnity in concision of the bacterial cell wall. Crit Rev Biochem and Molecular Biol 52: 503–542. doi: 10.1080/10409238.2017.1337705 28644060 PMC6102726

[18] Lee M, Hesek D, Llarrull LI, Lastochkin E, Pi H, Boggess B, Mobashery S (2013) Reactions of all *Escherichia coli* lytic transglycosylases with bacterial cell wall. J Am Chem Soc 135: 3311–3314. 23421439 10.1021/ja309036q PMC3645847

[19] Scheurwater E, Reid CW, Clarke AJ (2008) Lytic transglycosylases: Bacterial space-making autolysins. Int J Biochem Cell Biol 40: 586–591. doi: 10.1016/j.biocel.2007.03.018 17468031

[20] Park J.T, Uehara T (2008) How bacteria consume their own exoskeletons (turnover and recycling of cell wall peptidoglycan). Microbiol Mol Biol Rev 72: 211–227. doi: 10.1128/MMBR.00027-07 18535144 PMC2415748

[21] Yunck R, Cho H, Bernhardt TG (2016) Identification of MltG as a potential terminase for peptidoglycan polymerization in bacteria. Mol Microbiol 99: 700–718. doi: 10.1111/mmi.13258 26507882 PMC4752859

[22] Yakhnina AA, Bernhardt TG (2020) The Tol-Pal system is required for peptidoglycan-cleaving enzymes to complete bacterial cell division. Proc Natl Acad Sci USA 117: 6777–6783. doi: 10.1073/pnas.1919267117 32152098 PMC7104345

[23] Cho H, Uehara T, Bernhardt TG (2014) Beta-lactam antibiotics induce a lethal malfunctioning of the bacterial cell wall synthesis machinery. Cell 6:1300–1311. doi: 10.1016/j.cell.2014.11.017 25480295 PMC4258230

[24] Weaver AI, Alvarez L, Rosch KM, Ahmed A, Wang GS, van Nieuwenhze MS et al (2022) Lytic transglycosylases mitigate periplasmic crowding by degrading soluble cell wall turnover products. elife 11:73178. 10.7554/eLife.73178 PMC8820737 35073258

[25] Weaver AI, Taguchi A, Dorr T (2023) Masters of misdirection: peptidoglycan glycosidases in bacterial growth. J Bacteriol 205: 00428–22. doi: 10.1128/jb.00428-22 36757204 PMC10029718

[26] Bateman A, Bycroft M (2000) The structure of a LysM domain from E. coli membrane-bound lytic murein transglycosylase D (MltD). J Mol Biol 299: 1113–1119. doi: 10.1006/jmbi.2000.3778 10843862

[27] Hsu PC, Chen CS, Wang S, Hashimoto M, Huang WC, Teng CH (2020) Identification of MltG as a Prc protease substrate whose dysregulation contributes to the conditional growth defect of Prc-deficient *Escherichia coli*. Front Microbiol 11:1–16. 32973722 10.3389/fmicb.2020.02000 PMC7481392

[28] Battesti A, Majdalani N, Gottesman S (2011) The RpoS-mediated general stress response in *Escherichia coli*. Ann Rev Microbiol 65: 189–213. 21639793 10.1146/annurev-micro-090110-102946 PMC7356644

[29] Weber H, Polen T, Heuveling J, Wendisch VF, Hengge R (2005) Genome-wide analysis of the general stress response network in *Escherichia coli*: σ^S^-dependent genes, promoters and sigma factor selectivity. J Bacteriol 187: 1591–1603. 15716429 10.1128/JB.187.5.1591-1603.2005 PMC1063999

[30] Ellermeier CD, Janakiraman A, Slauch JM (2002) Construction of targeted single copy lac fusions using λ Red and FLP-mediated site-specific recombination in bacteria. Gene 290: 153–161. 12062810 10.1016/s0378-1119(02)00551-6

[31] Kitagawa M, Ara T, Arifuzzaman M, Loka-Nakamichi T, Inamoto E, Toyonaga H et al (2005) Complete set of ORF clones of *Escherichia coli* ASKA library (a complete set of E. coli K-12 ORF archive): unique resources for biological research. DNA Res. 12(5): 291–299. 16769691 10.1093/dnares/dsi012

[32] Wientjes FB, Pas E, Taschner PEM, Woldringh CL (1985) Kinetics of uptake and incorporation of meso-diaminopimelic acid in different *Escherichia coli* strains. J Bacteriol 164: 331–337. 3900040 10.1128/jb.164.1.331-337.1985 PMC214248

[33] Martinez-Bond EA, Soriano BM, Williams AH (2022) The mechanistic landscape of lytic transglycosylase as targets for antibacterial therapy. Curr Opin Struct Biol 77: 102480. doi: 10.1016/j.sbi.2022.102480 36323133

[34] Liu X, den Blaauwen T (2023) NlpI-Prc proteolytic complex mediates peptidoglycan synthesis and degradation via regulation of hydrolases and synthases in *Escherichia coli*. Int J Mol Sci 24, 16355 38003545 10.3390/ijms242216355 PMC10671308

[35] Som N. and Reddy M. (2023) Cross-talk between phospholipid synthesis and peptidoglycan expansion by a cell wall hydrolase. Proc. Natl. Acad. Sci. U. S. A. 120, e2300784120 doi: 10.1073/pnas.2300784120 37276399 PMC10268279

[36] Miller J.H (1992) A short course in bacterial genetics. A laboratory manual and handbook for Escherichia coli and related bacteria. CSHL Press.

[37] Baba T, Ara T, Hasegawa M, Takai Y, Okumura Y, Baba M, et al (2006) Construction of *Escherichia coli* K-12 in-frame, single-gene knockout mutants: the Keio collection. Mol Syst Biol 2: 2006–008. 10.1038/msb4100050 PMC1681482 16738554

[38] Sharan SK, Thomason LC, Kuznetsov SG, Court DL (2009) Recombineering: A homologous recombination-based method of genetic engineering. Nat Protoc 4: 206–223. doi: 10.1038/nprot.2008.227 19180090 PMC2790811

[39] Uzzau S, Figueroa-Bossi N, Rubino S, Bossi L (2001) Epitope tagging of chromosomal genes in *Salmonella*. Proc Natl Acad Sci USA 98: 15264–15269. 11742086 10.1073/pnas.261348198 PMC65018

