# Supplementary material for: Glycan strand cleavage by a lytic transglycosylase, MltD contributes to the expansion of peptidoglycan in Escherichia coli
Source: PLoS Genet. 2024 Feb 29;20(2):e1011161. doi: 10.1371/journal.pgen.1011161 (PMC10931528; doi:10.1371/journal.pgen.1011161)
Supplement: S1 Text — Table A. List of strains used in this study. Table B. List of plasmids used in this study. (DOCX) [file pgen.1011161.s001.docx]

**Glycan strand cleavage by a lytic transglycosylase, MltD contributes to the expansion of peptidoglycan in *Escherichia coli***

Moneca Kaul^1,2^, Suraj Kumar Meher^1,2^, Krishna Chaitanya Nallamotu^1,2^, Manjula Reddy^1,2*^

^1^CSIR-Centre for Cellular and Molecular Biology, Hyderabad, India

^2^Academy of Scientific and Innovative Research (AcSIR), Ghaziabad, India

Short title: Role of MltD in peptidoglycan expansion of *E. coli*

*manjula@ccmb.res.in

**Supporting Materials and Methods:**

**Plasmid constructions:**

The genomic DNA from the WT strain, MG1655 was used as a template to amplify desired gene. PCR amplification was done using NEB Q5 high fidelity master mix. The clones that were obtained were confirmed by sequence analysis.

**pMK1:** This plasmid is a pTrc99a derivative carrying a cloned *mltD* gene under the *trc* promoter. *mltD* gene was cloned without its ribosome binding site using the following forward and reverse primers: 5’CGAGCTCATGAAGGCAAAAGCGATATTAC3’ and 5’CGGGATCCTCAGGAATCTGGCATGTTGTTG3’.

The resulting amplified DNA fragment was cloned using SacI-BamHI sites (underlined in the primer sequence) in a cloning vector pTrc99a to obtain pMK1.

**pMK2 and pMK3:** These are derivatives of pMK1 which have active site mutations in the *mltD* gene. For the generation of site directed variants of *mltD,* a three-step PCR was performed. For this, two primers that are complimentary to each other were synthesized with the desired mutation at the center. In the first PCR, N-terminal fragment of *mltD* gene was amplified using the forward primer used for pMK1 construction and the reverse primer containing the desired mismatch. In the second PCR, C-terminal fragment of *mltD* gene was amplified using forward mismatch containing primer and reverse primer that was used for pMK1 construction. The desired mismatches code for an alanine and lysine instead of a glutamate residue at position 125 (E125). The catalytic residue E125 was chosen based on PROSITE database, derived from sequence homology (Uniprot ID- P0AEZ7, P0AEZ8).

Primer sequence for making E125A is as follows (The changed codons are indicated in bold):

**pMK2** **(E125A)**. 5’ GGTACTACTACCCATAGTG**GCG**AGCGCTTTTGATCCTCACG 3’ and 5’ CGTGAGGATCAAAAGCGCT**CGC**CACTATGGGTAGTAGTACC 3’

Sequence for making E125K is as follows:

**pMK3 (E125K).**

5’ AACTGGTACTACTACCCATAGTG**AAA**AGCGCTTTTGATCCTCACGCAAC 3’ and 5’ GTTGCGTGAGGATCAAAAGCGC**TTT**TCACTATGGGTAGTAGTACCAGTTC 3’

The first round of PCR was done using the common forward and SDM reverse and SDM forward and common reverse primer for each of the mutants to obtain two fragments. In the third step, these fragments were mixed in 1:1 molar ratio and used as template for another round of PCR using the common forward and reverse primers. The final PCR fragment was digested with SacI and BamHI and cloned into pTrc99a digested with the same enzymes. The plasmids pMK2 and pMK3 were confirmed for the mutations by sequencing.

**pMK4:** This is a variant of pMK1 carrying *mltD* gene without LysM domains. Sequence was selected based on PROSITE-ProRule annotation of LysM domains. Truncated *mltD*^1-340^ without *lysM* domains was cloned in pTrc99a. The forward primer was same as the one used for constructing pMK1 and the reverse primer sequence is 5’GGGATCCTCAGCTGTTAAGCGGCGTATTGTC

3’ containing the BamHI site. The PCR fragment was digested with SacI and BamHI and cloned in pTrc99a vector digested with the same enzymes. The truncation was confirmed by sequencing.

**pMK5:** *mltD* (without its promoter and RBS) having a C-terminal 1XFlag tag was constructed using the common forward primer used for construction of pMK1 and the reverse primer is of the following sequence: 5’CGGGATCCTTACTTGTCATCGTCATCCTTGTAATCGGAATCTGGCATGTTGTTG 3’ containing BamHI site. The PCR fragment was digested with SacI and BamHI and cloned in pTrc99a vector digested with the same enzymes. The fusion was confirmed by sequencing.

**pMK6:** The *mltD*^18-452^ fragment lacking the N-terminal signal sequence (His-MltD) was cloned into pET28b vector using NdeI-BamHI sites for overexpression and purification. The forward and reverse primer sequences are as follows: 5’ GGAATTCCATATGAGTACCGGCAACGTTCAACAG 3’ and 5’ CGGGATCCTCAGGAATCTGGCATGTTGTTG 3’.

**Strain constructions:**

**Construction of deletion alleles**: Deletion strains used in the study were sourced from the Keio collection [1]. The presence of gene deletion was validated by PCR followed by sequence analysis. Deletions were introduced into the desired strains by P1 transductions. Wherever required, the Kan gene was flipped out using pCP20 plasmid.

**Construction of *mltD*-Flag, *mltA*-Flag and *mltF*-Flag fusions:** The 3xFlag epitope tagging of *mltD*, *mltA* or *mltF* genes was done at the 3′ end at their native chromosomal locus as previously described [3]. Hybrid primers with regions homologous to the chromosomal loci and Flag-Kan cassette of pSUB11 were used. The regions homologous to chromosome are underlined and the region homologous to pSUB11 are italicized. The primer sequences are as follows:

***mltD*-3x Flag:**

5’AAGCTGACGTTGTTTGTGAAAAACAACAACATGCCAGATTCC*GACTACAAAGACCATGACGG* 3’ and 5’AAGGCACCGGGGGAATCGGTGCCTTTTTATTATCTGGTTTG*CATATGAATATCCTCCTTAG* 3’

***mltA*-3x Flag:**

5’GTGCTGAAAACCGCCCCGGGCGCAGGTAACGTCTTTAGCGGC*GACTACAAAGACCATGACCGG* 3’ and

5’TCACCCTGTCATATCCGTAAAAACGGCATACAGAATATCACACA*CATATGAATATCCTCCTTAG* 3’

***mltF*-3x Flag:**

5’TCTCTGCTGTTTTCCAGGAAAGGGAGTGAAGAGAAACAAAAT*GACTACAAAGACCATGACGG* 3’ and

5’CCAGGAAATTAAAGCGCAGAAAAAAGCGCAATCCTCGACGGA*CATATGAATATCCTCCTTAG* 3’

These primers were used to amplify the 3xFlag sequence from the plasmid pSUB11 with a Kan^R^ marker flanked by FRT (flippase recognition target) sites. The PCR products were electroporated into DY378, and colonies were obtained at 30°C on LB plates containing 25 μg/ml Kan. The gene-Flag-Kan^R^ region was transferred from DY378 into MG1655 by P1 transduction. These constructs were confirmed by PCR amplification, sequencing and western blotting using Flag antibodies. When required, the Kan^R^ cassette was flipped out using pCP20, and the Kan-sensitive (Kan^S^) derivatives of *mltD*-3xFlag were made.

**Construction of P*_mltD_*:: *lacZY-*Kan strain:** The *mltD*::Kan deletion strain of the Keio collection was converted into *lacZY-*Kan fusion as described earlier [4]. Initially, the deletion mutation was transferred into MC4100 strain (a strain carrying deletion of complete Lac operon) by P1 transduction. Next, the plasmid pCP20, encoding Flp recombinase was introduced into this strain to flip out the Kan^R^ determinant at 30°C. Subsequently, into the Kan^S^ derivatives carrying pCP20, an R6K based plasmid, pKGE137, encoding a promoter-less *lacZY* cassette along with Kan^R^ marker flanked by FRT sites was introduced. Kan^R^ colonies were selected on plates supplemented with X-Gal, and the presence of *lacZ*-Kan cassette at *mltD* locus was confirmed by PCR and sequencing. The P*_mltD_*:: *lacZY*-Kan fusion was then transferred into MC4100 or its *ΔrpoS* deletion derivative.

**Supplementary methods:**

**Protein overexpression.** Overexpression plasmids encoding NlpI, Prc, MepS, MepM were described earlier [5,6]. MltD overexpression plasmid was constructed in this study. Overexpression of proteins was done using T7 RNA polymerase-based system as described earlier [5,6]. Overexpression of His-MltD was done in BL21 (λDE3) and a single transformant was grown overnight, and subcultured (1:100 ratio) into fresh LB broth containing respective antibiotics and grown till an OD_600_ of 0.6 with shaking at 37⁰C. Expression of MltD was induced by adding 50 µM IPTG (optimally standardized to achieve the highest amount of soluble protein) and grown further for 2 h at 37⁰C. Cells were centrifuged and the pellet was then processed for purification of the expressed protein.

For the purification of His-MepS, Prc-His, MepM-His and NlpI-His, the plasmids were transformed into BL21 (λDE3) ∆*nlpI* strain and processed as described above with minor modifications:

His-MepS: Induced with 200 µM IPTG at 37⁰C for 3 h.

MepM-His: Induced with 100 µM IPTG at 37⁰C for 3 h.

Prc-His: Induced with 500 µM IPTG at 25⁰C for 5 h.

NlpI-His: Induced with 500 IPTG at 37⁰C for 3 h.

**Protein purification.** MepS, MepM, NlpI and Prc were purified as described earlier [5,6]. For MltD, the harvested cells were resuspended in 20 ml of lysis buffer (50 mM Tris pH8.0, 300 mM NaCl, 10 mM imidazole) and further lysed by sonication for 15-20 min (20% Amplitude; 5-sec on-off cycle). The overexpressed proteins were soluble and did not require any detergent for solubilization. Cell debris was then removed by centrifugation at 30,000X RCF for 20 min at 4⁰C and the supernatant was mixed with 0.5 ml of Ni2+- NTA agarose (Qiagen) at 4⁰C for 1 h. This mixture was loaded into empty glass columns (Bio-Rad) and the supernatant was then allowed to pass through to retain the Ni-NTA beads inside the column. The beads were then washed with 20 ml of wash buffer-I (50 mM Tris pH 8.0, 300 mM NaCl, 30 mM imidazole, 1% Triton-X-100), then with 20 ml of wash buffer-II (50 mM Tris pH 8.0, 300 mM NaCl, 50 mM imidazole). Bound proteins were eluted with 10 ml of elution buffer (50 mM Tris pH 8.0, 300 mM NaCl, 250 mM imidazole). The eluted protein was buffer exchanged using a 30 kDa cut-off centrifugal membrane filter (Millipore) with 2X storage buffer (100 mM Tris pH8.0, 200 mM NaCl, 2 mM DTT) and concentrated. An equal volume of 100% glycerol (molecular grade) was added and protein sample was then stored at -30⁰C. A small aliquot was run on SDS-PAGE to evaluate the purity of the protein.

**Pull down assays.** Pull downs (or co-purifications) were performed as described earlier [6]. Strains were grown overnight and next day, diluted 1:100 into 150 ml of LB and allowed to grow until OD_600_ of 0.8. Cells were recovered by centrifugation, and the pellet was resuspended in 20 ml of lysis buffer (50 mM of Tris-Cl, 100 mM NaCl, 20% glycerol, 1% Triton X 100, 250 µg/ml lysozyme, 1X protease inhibitor, 20 units of DNase, 20 µg/ml of RNase and 10 mM imidazole; pH 8.0) and incubated in ice for 2 h. The mixture was sonicated, and the lysate was stirred overnight with glass beads at 4°C to solubilize the membrane proteins. Next day, the insoluble material of the lysate was removed by centrifugation at 15000 x g for 30 min. 100 µl from the supernatant was set aside as input fraction. To the rest of the supernatant, 300 µl of Ni-NTA agarose beads were added and mixed for 2 h at 4°C. The agarose beads were washed twice with wash buffer I (50 mM Tris-Cl, 100 mM NaCl, 1% Triton X 100, 20% glycerol and 20 mM imidazole; pH 8.0) and II (50 mM Tris-Cl, 100 mM NaCl, 20% glycerol and 50 mM imidazole; pH 8.0). The bound proteins were eluted using 250 µl elution buffer I (50 mM Tris-Cl, 100 mM NaCl, and 150 mM imidazole; pH 8.0) and II (50 mM Tris-Cl, 100 mM NaCl, 300 mM imidazole; pH 8.0). The input samples along with the elution fractions were subjected to SDS-PAGE and the proteins were detected by western blotting.

**β-galactosidase assay**. Assays were performed as described previously [7] using MC4100 *mltD::lacZY* strain or its derivatives. Strains were grown overnight in LB medium, and next day diluted 1:50 into fresh LB medium and grown till either O.D_600_ of 0.5 or 3.0. For 3 OD cultures, cells were diluted 1:6 and processed similarly as described [7]. ONPG was used as a substrate.

**Table A. List of strains used in the study**

| **Strain** | **Genotype** | **Source/ Reference** |
| --- | --- | --- |
| MG1655 | *rph*1 *ilvG rfb*-50 | Lab collection |
| DH5α | F^–^ *hsdR17 deoR recA1 endA1 phoA supE44 thi-1 gyrA96 relA1* Δ*(lac-argF)U169 φ80dlacZ* Δ*M15* | Lab collection |
| BW25113 | l*acI*^q^ *rrnB3* ∆*lacZ4787* ∆(*araBAD*)567 ∆(*rhaBAD*)568 *hsdR*514 | [1] |
| DY378 | W3110 *λC1857* ∆(cro-bioA) | [2] |
| MC4100 | F− *araD139* Δ*argF*-*lac169* λ− e14−*flhD5301* *relA1* *rpsL150* *rbsR22* *deoC1* | Lab collection |
| BL21(λDE3) | *ompT rB- mB-* (*PlacUV*5*::T*7*-*1*)* | Novagen |
| MR510 | Δ*mepS* Δ*mepM*/ pMN83 | [5] |
| MR508 | Δ*mepS* Δ*mepM* Δ*mepH*/ pMN83 | [5] |
| MR810 | *mepS*::*frt* | [5] |
| MK01 | Δ*mepS* Δ*mepM::Cm* | This study |
| MK02 | MK01 *prc** | This study |
| MK03 | MK01 *nlpI** | This study |
| MK04 | MK01 Δ*prc::*Kan | This study |
| MK05 | MK01 Δ*nlpI::*Kan | This study |
| MK06 | ∆*mepS* ∆*mepM* ∆*nlpI* (Kan^S^)/ pMN83 | Lab collection |
| MK07 | MK06 Δ*mepA*::Kan | This study |
| MK08 | MK06 Δ*mepH*::Kan | This study |
| MK09 | MK06 Δ*dacB*::Kan | This study |
| MK10 | MK06 Δ*pbpG*::Kan | This study |
| MK11 | MK06 Δ*ampH*::Kan | This study |
| MK12 | MK06 Δ*mltA*::Kan | This study |
| MK13 | MK06 Δ*mltB*::Kan | This study |
| MK14 | MK06 Δ*mltC*::Kan | This study |
| MK015 | MK06 Δ*mltD*::Kan | This study |
| MK016 | MK06 Δ*mltE*::Kan | This study |
| MK017 | MK06 Δ*mltF*::Kan | This study |
| MK018 | MK06 Δ*mltG*::Kan | This study |
| MK019 | MK06 Δ*digH*::Kan | This study |
| MK020 | MK06 Δ*slt*::Kan | This study |
| MK022 | MR508 Δ*prc*::Kan | This study |
| MK023 | MR508 Δ*nlpI*::Kan | This study |
| MK024 | MK04 Δ*mltD*::Kan | This study |
| MK025 | *mltD*-Flag-frt | This study |
| MK026 | Δ*nlpI*::*frt* MK025 | This study |
| MK027 | Δ*prc*::*frt* MK025 | This study |
| MK028 | Δ*nlpI* Δ*prc::frt* MK025 | This study |
| MK038 | *mepM::frt*/ pTrc99a | This study |
| MK039 | *mepM::frt*/ pMK1 | This study |
| MK040 | MG1655 P_lac_::*mltD* | This study |
| MK041 | MR810 P_lac_::*mltD* | This study |
| MK042 | *mltD*::*frt* | This study |
| MK043 | MR810 *mltD*::*frt* | This study |
| MK044 | MR510 Δ*mltD*::Kan | This study |
| MK045 | MG1655 Δ*lysA*::Kan | This study |
| MK046 | MR810 Δ*lysA*::Kan | This study |
| MK047 | MK042 Δ*lysA*::Kan | This study |
| MK048 | MK043 Δ*lysA*::Kan | This study |
| MK049 | *mepM*::*frt* Δ*lysA*::Kan | This study |
| MK050 | MK049 *mltD*::*frt* Δ*lysA*::Kan | This study |
| MK051 | *mltA*-3xFlag-Kan | This study |
| MK052 | MK051 Δ*nlpI*::*frt* | This study |
| MK053 | MK051 Δ*prc*::*frt* | This study |
| MK054 | *mltF*-Flag-Kan | This study |
| MK055 | MK054 Δ*nlpI*::*frt* | This study |
| MK056 | MK054 Δ*prc*::*frt* | This study |
| MK057 | MK025 Δ*rpoS*::Kan | This study |
| MK058 | MK026 Δ*rpoS*::Kan | This study |
| MK059 | MK027 Δ*rpoS*::Kan | This study |
| MK060 | MK025/pTrc99a | This study |
| MK061 | MK058/ pMK5 | This study |
| MK062 | MK026/pMK5 | This study |
| MK063 | MK026 ∆r*poS*::Kan/pMK5 | This study |
| MK064 | MK027/pMK5 | This study |
| MK065 | MK027 ∆r*poS*::Kan/pMK5 | This study |
| MK066 | MC4100 P*_mltD_::LacZY*-Kan | This study |
| MK067 | MC4100 Δ*rpoS* P*_mltD_::LacZY*-Kan | This study |
| MK071 | MK026 *nlpI*::*frt prc*-HA-Cm/ pMN218 | This study |
| MK072 | MK025 *prc*-HA-Cm *mepS*::*frt*/ pMN217 | This study |
| MK073 | MK025 *prc*-HA-Cm *mepS*::*frt nlpI*::*frt*/ pMN217 | This study |
| MK083 | Δ*mepS* Δ*mepM::*Cm Δ*prc* | This study |
| MK084  MK085  MK086  MK087  MK088  MK089  MK090  MK091  MK092  MK093  MK094  MK095  MK096  MK097  MK098  MK099  MK100  MK101  MK102  MK103  MK104  MK105 | Δ*mepS* Δ*mepM::*Cm Δ*prc mltD::Kan*  MR810/ pTrc99a  MR810/ pMK1  MR810/ pMK2 MR810/ pMK3  MR810/ pMK4  MR510/ pTrc99a  MR510/ pMK1  MR510/ pMK2  MR510/ pMK3  MK091/ Δ*mrcA*::Kan  MK091/ Δ*mrcB*::Kan  MK091/ Δ*lpoA*::Kan  MK091/ Δ*lpoB*::Kan  MR508/ pTrc99a  MK508/ pMK1  MR508/ pMK2  MR508/ pMK3  MK099/ Δ*mrcA*::Kan  MK099/ Δ*mrcB*::Kan  MK099/ Δ*lpoA*::Kan  MK099/ Δ*lpoB*::Kan | This study  This study  This study  This study  This study  This study  This study  This study  This study  This study  This study  This study  This study  This study  This study  This study  This study  This study  This study  This study  This study  This study |

**Table B. List of plasmids used in the study**

| **Plasmids** | **Relevant Features** | **Source/ Reference** |
| --- | --- | --- |
| pCA24N | Cm^R^, *lacI*^q^,P_T_*_5-lac_* | [8] |
| pCA24N-*mltA* | Cm^R^, *lacI*^q^,P_T5_*_-lac_*_::_*mltA* | [8] |
| pCA24N-*mltC* | Cm^R^, *lacI*^q^,P_T5_*_-lac_*_::_*mltC* | [8] |
| pCA24N-*mltD* | Cm^R^, *lacI*^q^,P_T5_*_-lac_*_::_*mltD* | [8] |
| pCA24N-*mltE* | Cm^R^, *lacI*^q^,P_T5_*_-lac_*_::_*mltE* | [8] |
| pCA24N-*mltF* | Cm^R^, *lacI*^q^,P_T5_*_-lac_*_::_*mltF* | [8] |
| pCA24N-*mltG* | Cm^R^, *lacI*^q^,P_T5_*_-lac_*_::_*mltG* | [8] |
| pCA24N-*digH* | Cm^R^, *lacI*^q^,P_T5_*_-lac_*_::_*digH* | [8] |
| pCA24N-*slt* | Cm^R^, *lacI*^q^,P_T5_*_-lac_*_::_s*lt* | [8] |
| pTrc99a | ColE1, Amp^R^, *lacIq*, T7*lac* | Lab collection |
| pCP20 | pSC101(Ts), Amp^R^, Cm^R^, Flp | Lab collection |
| pMN83 | pBAD33-*mepS* | [5] |
| pET28b | T7 *lac* promoter; Kan^R^ | Lab collection |
| pMN219 | pET21b-*prc*^23-682^-His | [6] |
| pMN208 | pET21b-*nlpI^19^*^-294^-His | [6] |
| pMN218 | pBAD18-*nlpI*-His, Amp^R^ | [6] |
| pMN217 | pBAD18-*mepS*-His, Amp^R^ | [6] |
| pMK1 | pTrc99a-*mltD* | This study |
| pMK2 | pTrc99a-*mltD*_E125A_ | This study |
| pMK3 | pTrc99a-*mltD*_E125K_ | This study |
| pMK4 | pTrc99a-*mltD*^1-340^ | This study |
| pMK5 | pTrc99a-*mltD-*Flag | This study |
| pMK6 | pET28b-6X His-*mltD*^18-452^ | This study |

**Supplemental References:**

1. Baba T, Ara T, Hasegawa M, Takai Y, Okumura Y, Baba M, et al (2006) Construction of *Escherichia coli* K-12 in-frame, single-gene knockout mutants: the Keio collection. Mol Syst Biol 2: 2006-008.
2. Sharan SK, Thomason LC, Kuznetsov SG, Court DL. 2009. Recombineering: A homologous recombination-based method of genetic engineering. Nat Protoc 4: 206–223.
3. Uzzau S, Figueroa-Bossi N, Rubino S, Bossi L. 2001. Epitope tagging of chromosomal genes in *Salmonella*. Proc Natl Acad Sci USA 98 Proc Natl Acad Sci USA 98: 15264–15269.
4. Ellermeier CD, Janakiraman A, Slauch JM. 2002. Construction of targeted single copy lac fusions using λ Red and FLP-mediated site-specific recombination in bacteria. Gene 290: 153-161.
5. Singh SK, Saisree L, Amrutha RN, Reddy M (2012) Three redundant murein endopeptidases catalyse an essential cleavage step in peptidoglycan synthesis of *Escherichia coli* K12. Mol Microbiol 86:1036–1051.
6. Singh SK, Parveen S, SaiSree L, Reddy M (2015) Regulated proteolysis of a cross-link-specific peptidoglycan hydrolase contributes to bacterial morphogenesis. Proc Natl Acad Sci USA 112:10956–10961.
7. Miller J.H. 1992. A short course in bacterial genetics. A laboratory manual and handbook for *Escherichia coli* and related bacteria. CSHL Press.
8. Kitagawa M, Ara T, Arifuzzaman M, Loka-Nakamichi T, Inamoto E, Toyonaga H et al (2005) Complete set of ORF clones of *Escherichia coli* ASKA library (a complete set of *E. coli* K-12 ORF archive): unique resources for biological research. DNA Res. 12(5): 291–299.
