# Supplementary figures and images for: Glycan strand cleavage by a lytic transglycosylase, MltD contributes to the expansion of peptidoglycan in Escherichia coli
Source: PLoS Genet. 2024 Feb 29;20(2):e1011161. doi: 10.1371/journal.pgen.1011161 (PMC10931528; doi:10.1371/journal.pgen.1011161)

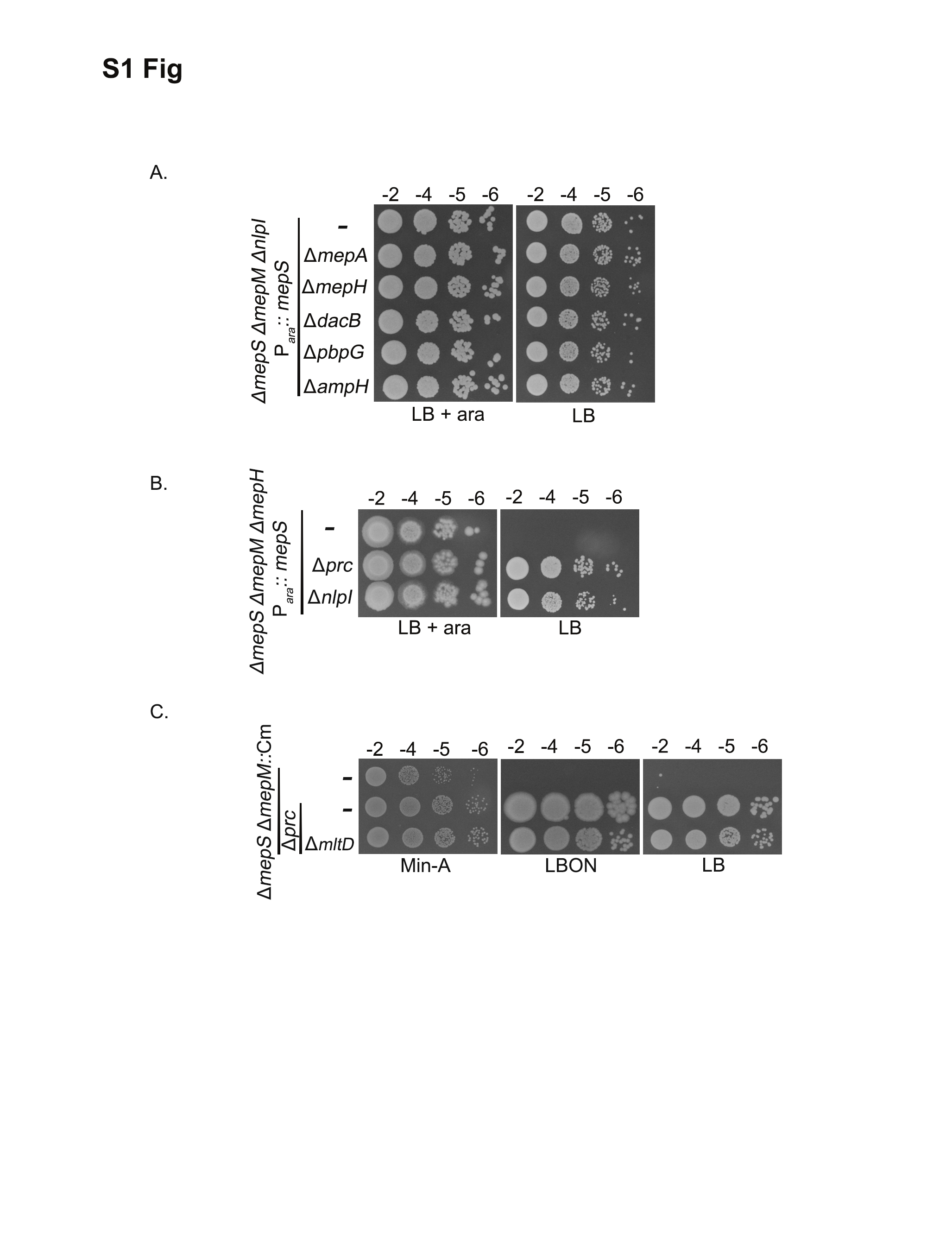

Supplement: S1 Fig — (A) Indicated strains carrying deletion of each of the known D,D-endopeptidases were tested for viability on LB with and without arabinose (0.2%). (B) Indicated strains and its mutant derivatives lacking nlpI or prc were tested for viability on LB with and without arabinose (0.2%). (C) ΔmepSM double mutant or its derivatives were grown overnight in MinA and their viability was tested on MinA, LBON (LB without NaCl) or LB plates. (TIF) [file pgen.1011161.s002.tif]

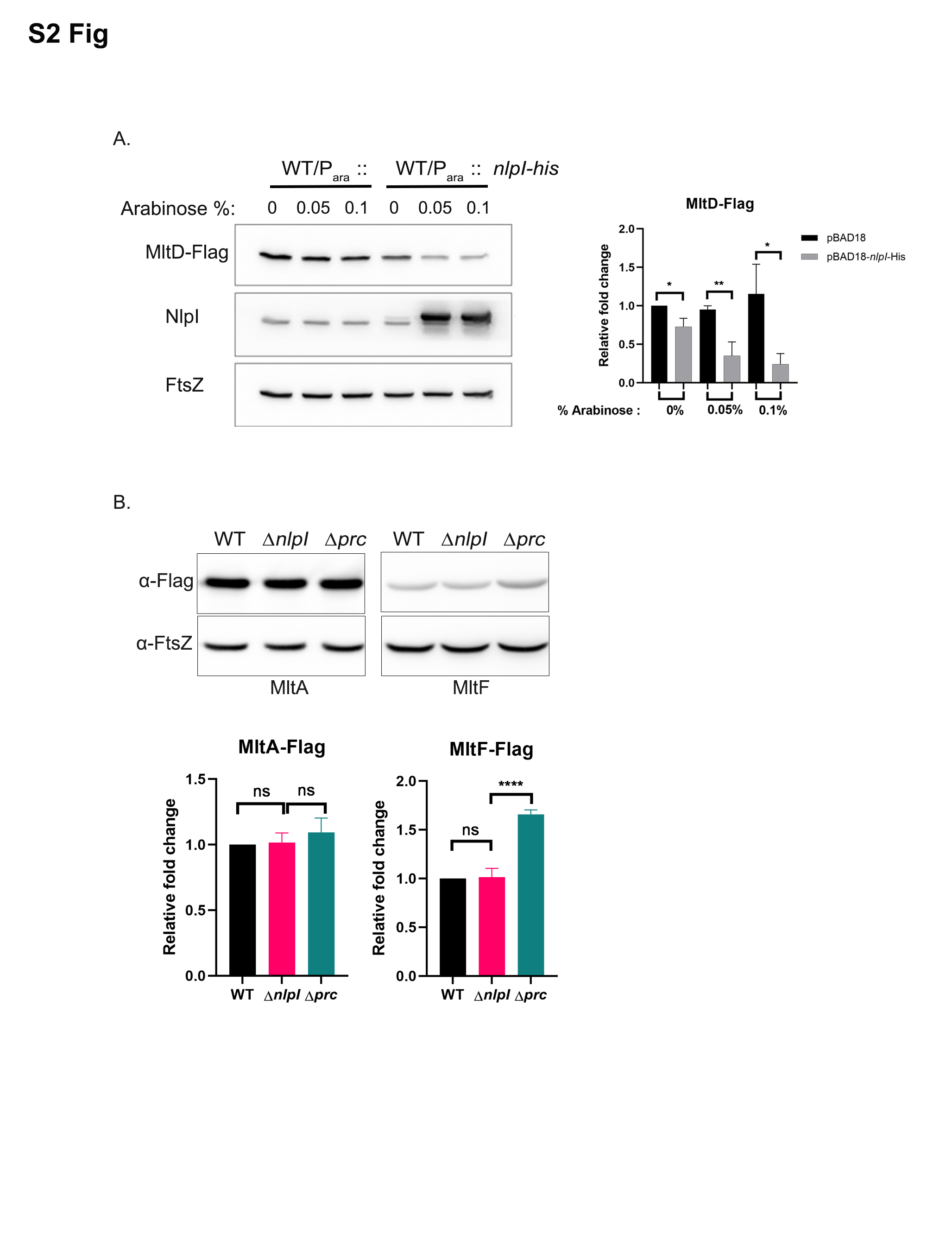

Supplement: S2 Fig — (A) Western blot showing the level of MltD-Flag in indicated strains. Cells were grown in LB supplemented with arabinose and fractions were collected between OD600 of 0.8–1.0. Normalized cell fractions were subjected to SDS-PAGE and analyzed by western blot. (B) WT and its mutant derivatives carrying mltA-Flag or mltF-Flag at their native chromosomal locus were grown in LB and processed as described above. Bar diagrams indicate the relative fold change of respective protein levels from three replicates; *, P <0.05; **, P <0.005; ****, P <0.0001; ns (not significant); n = 3. (TIF) [file pgen.1011161.s003.tif]

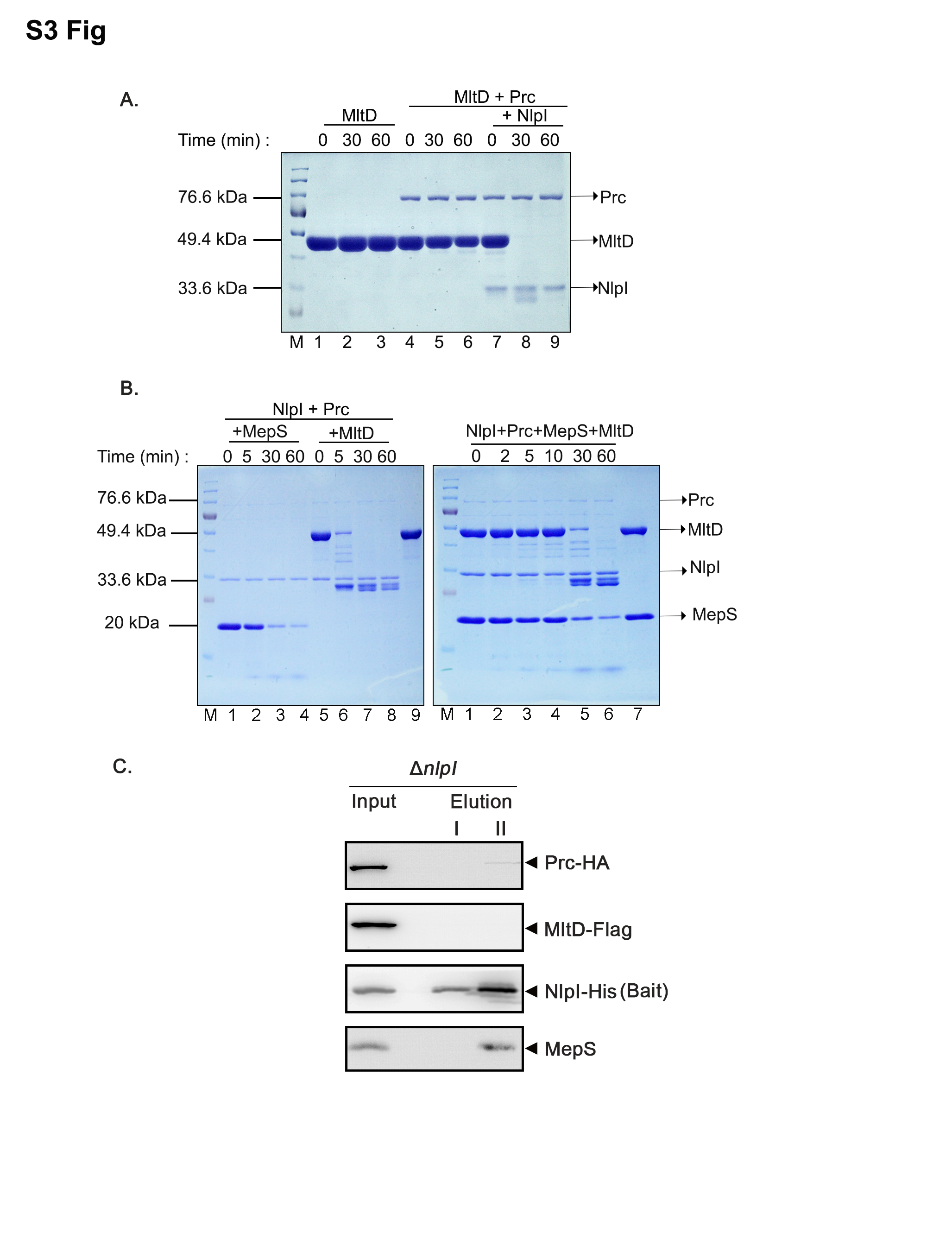

Supplement: S3 Fig — (A) In vitro degradation assay with purified MltD, Prc and NlpI proteins. Proteins were mixed in different combinations and incubated at 37°C followed by SDS-PAGE. Amounts of proteins used are as follows: MltD- 10 μg, NlpI- 1 μg, Prc- 0.4 μg. (B) In vitro degradation assay with purified MepS, MltD, Prc and NlpI proteins. Left panel shows degradation of MepS or MltD. Right panel shows the degradation of both MepS and MltD. (C) In vivo pull-down assay to check the interaction of NlpI with MltD. Plasmid borne NlpI-His was used as a bait to examine its interaction with MltD-Flag as described in SI. Prc-HA and MepS are used as positive controls. (TIF) [file pgen.1011161.s004.tif]

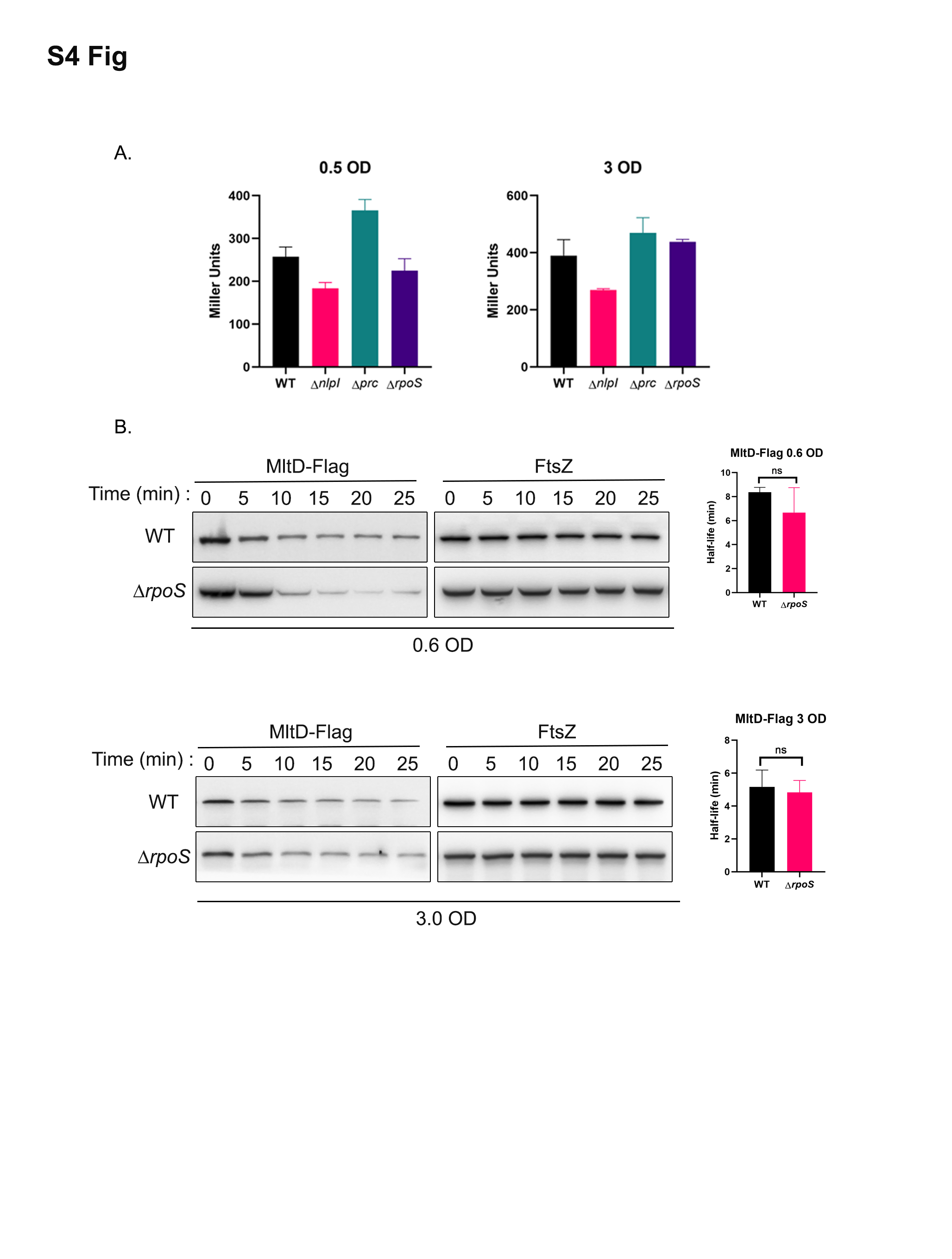

Supplement: S4 Fig — (A) β-galactosidase values of PmltD:: lacZY in WT and rpoS deletion mutant at OD600 of 0.6 and 3.0. Assays were performed as described in SI. Values are calculated as Miller units and indicated as bar graphs. (B) Half-life of MltD-Flag in the indicated strains was checked as follows: cells were grown in LB till OD600 of 0.6 or 3.0 and 300 μg/ml or 1 mg/ml of spectinomycin was added to block translation. Fractions were collected at indicated time points and were analysed by western blotting, as described in Materials and Methods. Error bars represent standard deviation. ns–not significant. FtsZ was used as a loading control. (TIF) [file pgen.1011161.s005.tif]

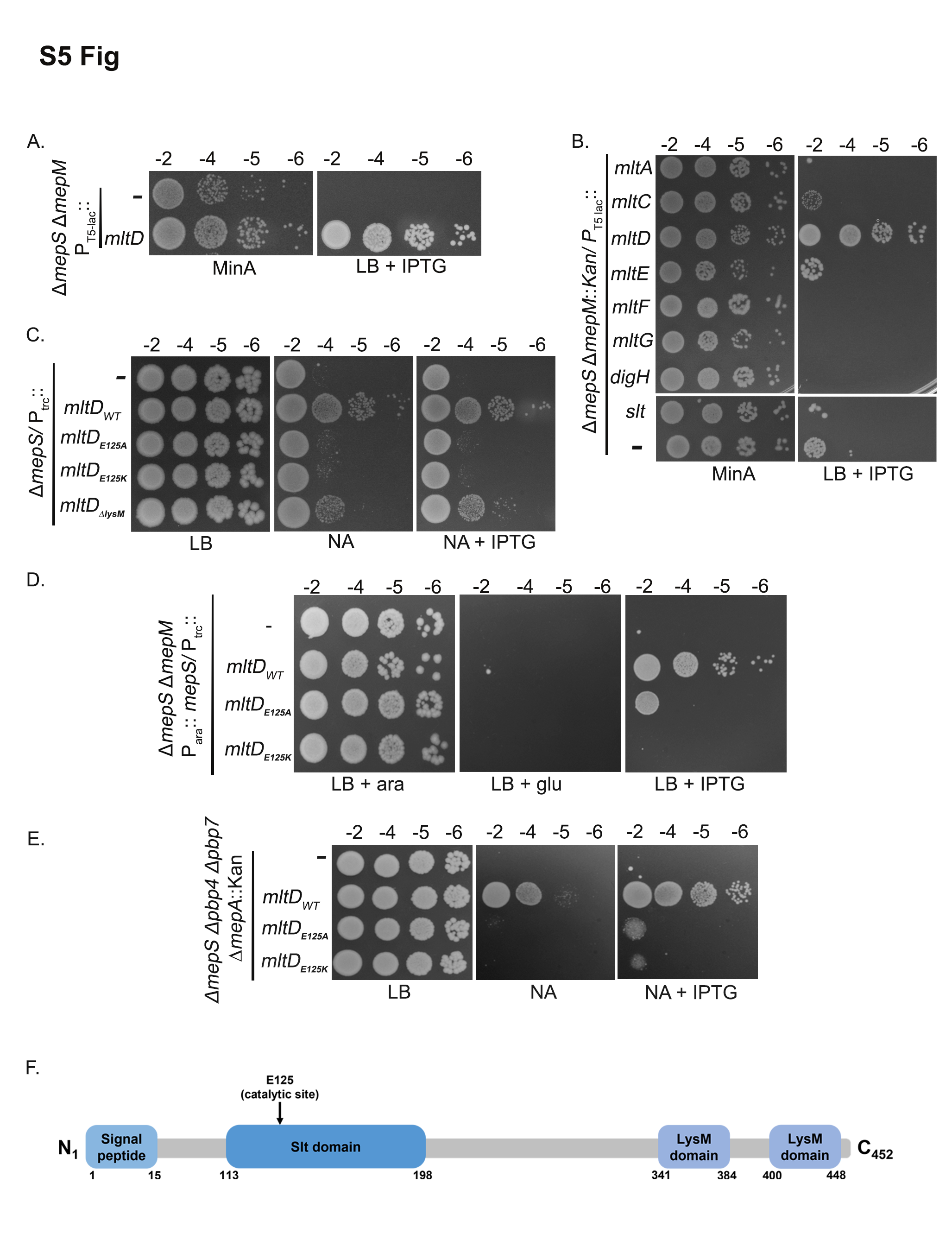

Supplement: S5 Fig — (A) Cells of mepS mepM mutant carrying pCA24N (ASKA empty vector) or pCA24N-mltD were grown overnight in MinA medium and viability was tested on LB plates with IPTG (25 μM). (B) Growth of indicated strains was checked on MinA or LB plates with IPTG (20 μM). pCA24N-MltB clone was not used in this experiment. (C, D, E) Indicated strains carrying pTrc99, pTrc99a-mltD or its derivatives were grown overnight in LB and viability was tested on indicated plates. NA plates had 10 μM IPTG whereas LB plates had 100 μM IPTG. (F) Depiction of structural features of MltD protein indicating its transglycosylase domain and two C-terminal LysM repeats. (TIF) [file pgen.1011161.s006.tif]

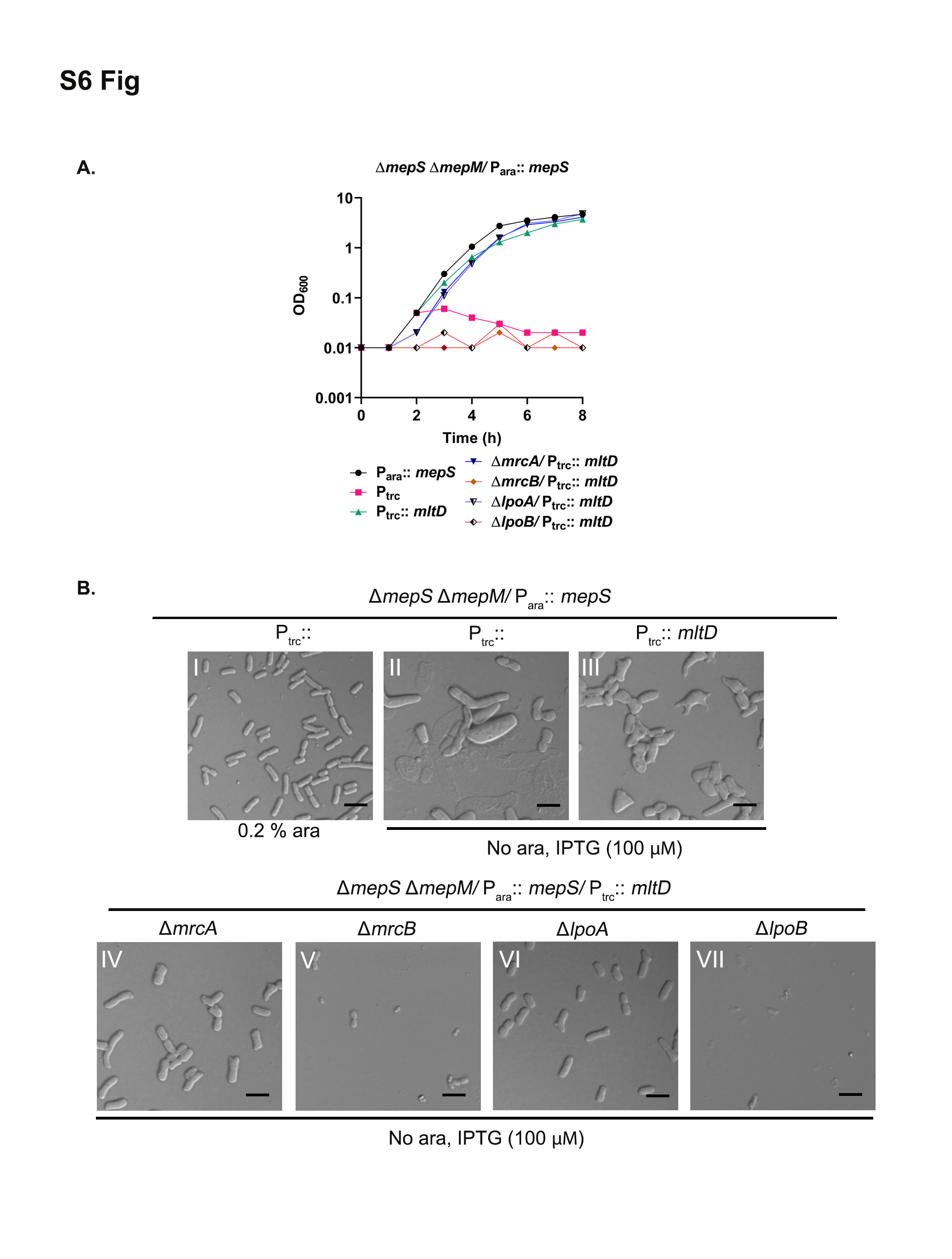

Supplement: S6 Fig — (A, B) Overnight grown cultures of the indicated strains were diluted 1:2500 into fresh LB containing appropriate inducers (0.2% arabinose or 100 μM IPTG) at 37°C and growth was monitored by OD600. Cells were collected after 3 h of growth and subjected to DIC microscopy as described in Materials and Methods. Scale bars represent 5 μm. (TIF) [file pgen.1011161.s007.tif]
